# *Artemisiae Argyi* exosome-like nanovesicles alleviate ALF by reducing oxidative stress and inhibiting inflammation through the TLR4/NLRP3/Nrf2 axis

**DOI:** 10.1186/s13020-026-01345-9

**Published:** 2026-02-16

**Authors:** Wenjie Zheng, Yue Su, Yan Tang, Kexin Yu, Runlin Lin, Yiyi Shan, Yuanyuan Wang, Louqin Fu, Jingjing Li

**Affiliations:** 1 Zhejiang Chinese Medical University, Hangzhou, 310053 China; 2 Clinical Research Institute, Zhejiang Key Laboratory of Tumor Molecular Diagnosis and Individualized Medicine, Zhejiang Provincial People’s Hospital, Affiliated People’s Hospital of Hangzhou Medical College, No. 158 Shangtang Road, Hangzhou, 310014 China; 3 Center for Rehabilitation Medicine, Rehabilitation and Sports Medicine Research Institute of Zhejiang Province, Department of Rehabilitation Medicine, Zhejiang Provincial People’s Hospital, Affiliated People’s Hospital of Hangzhou Medical College, Hangzhou, 310014 China

**Keywords:** ALF, PELNVs, Inflammatory responses, TLR4/NF-κB/NLRP3, MicroRNA

## Abstract

In recent years, plant-derived exosome-like nanovesicles (PELNVs) have attracted considerable research interest as emerging therapeutic adjuvants or delivery vehicles. This study aimed to develop exosome-like nanovesicles derived from *Artemisia argyi* (AELNVs) and evaluate their therapeutic potential in ameliorating LPS/D-GalN-induced acute liver failure (ALF) in mice. In our in vitro study, oral administration of AELNVs significantly ameliorates liver pathological damage, reduced systemic inflammatory cytokine levels, and improved survival in mice with ALF. We demonstrated that AELNVs inhibited the TLR4/NF-κB/NLRP3 axis and suppress hepatocyte apoptosis, while concurrently activating the Nrf2-CYP2A5 pathway to alleviate oxidative stress and hepatotoxicity. Analysis of hepatic chemokines revealed that AELNVs mitigated the hepatic immune response by suppressing the CCL5/7-CCR5 and CXCL9/10/11-CXCR3 chemokine axes. Furthermore, RNA-miRNA alignment analysis identified MIR2916 as a key mediator through which AELNVs modulated the TLR4 and its downstream signaling pathways. Collectively, these findings reveal the therapeutic potential of AELNVs as a nanotherapeutic agent for ALF.

**Graphical Abstract:**

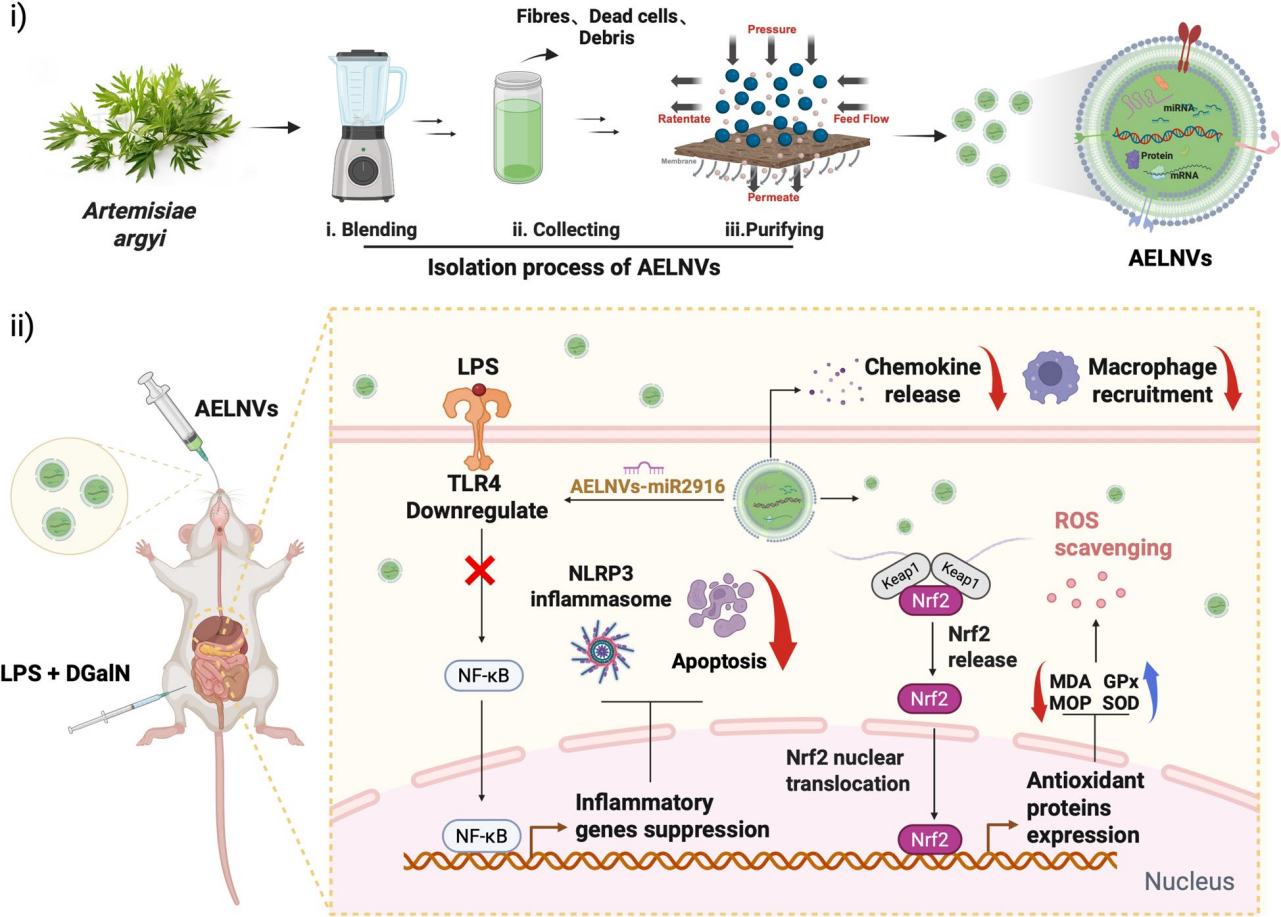

**Supplementary Information:**

The online version contains supplementary material available at 10.1186/s13020-026-01345-9.

## Introduction

Acute liver failure (ALF) is a critical clinical syndrome marked by profound hepatic dysfunction and elevated mortality rates. This condition presents a significant challenge to global public health and imposes considerable economic burdens. Currently, no effective pharmacological therapies are available for ALF, and liver transplantation remains the only established curative option; however, its widespread use is constrained by donor organ shortages and the risk of post-transplant immune rejection [1]. The etiology of ALF is heterogeneous, encompassing viral infections, metabolic disorders, and drug- or toxin-induced hepatotoxicity. While the precise pathogenesis remains incompletely understood, it is widely recognized that dysregulation of immune homeostasis, excessive oxidative stress, and acute hepatocyte injury play pivotal roles in disease progression [2]. Notably, extensive necrosis and apoptosis of hepatocytes lead to the release of pro-inflammatory cytokines and damage-associated molecular patterns (DAMPs), which trigger a robust systemic inflammatory response, exacerbate hepatic damage, and contribute to multiorgan dysfunction [3]. Therefore, a deeper understanding of the underlying pathophysiological mechanisms of ALF is crucial for the development of novel therapeutic interventions and the expansion of clinical treatment strategies.

*Artemisiae Argyi* (*A. argyi*) is a herb that has been extensively studied in traditional Chinese medicine., demonstrating significant potential for clinical applications across various domains. These include analgesia and antioxidant stress responses [4], antiviral defense and anti-tumor characteristics [5], as well as immunomodulation and anti-inflammatory [6]. Numerous studies indicated that various active components in *A. argyi* effectively alleviated multiple inflammation-related diseases by regulating key signaling pathways. *A. argyi* water extract exhibited potent anti-inflammatory effects, significantly suppressing LPS-induced NO concentration and the overexpression of inflammatory cytokines [7]; *A. argyi* essential oil reduced the production of toxic metabolites by modulating CYP2E1 levels, thereby alleviating APAP-induced oxidative stress and inflammation in animal models of liver injury [8]; *A. argyi* polysaccharide exerted anti-inflammatory effects in a permeation diarrhea model by inhibiting the TLR4/MyD88/NF-κB pathway to reduce the release of pro-inflammatory cytokine and increase anti-inflammatory factor secretion [9]; *A. argyi* polysaccharides AARP-1 and AARP-2 were found to possess immunomodulatory effects, significantly promoting macrophage proliferation and phagocytic activity by activating the TLR4/MAPK signaling pathway [10]. These studies systematically elucidated the anti-inflammatory and immunomodulatory effects of *A. argyi* through multi-component, multi-pathway synergistic mechanisms, ranging from molecular mechanisms to animal models. However, traditional extraction methods for Chinese herbal medicine suffer from issues such as disruption of the structural integrity of active components, low solubility of effective constituents, and poor bioavailability. Therefore, we need to explore new delivery forms for herbal medicines.

Plant-derived extracellular vesicle-like nanovesicles (PELNVs) have garnered significant attention in recent years as a promising alternative to mammalian cell-derived extracellular vesicles, owing to their unique biocompatibility, low immunogenicity, and scalable production potential [11]. PELNVs contain multiple bioactive components from the parent plant, including miRNAs, lncRNAs, glycosides, proteins, and hydrophobic small molecules as natural nanocarriers [12]. Their unique phospholipid bilayer structure enables the encapsulation and solubilization of hydrophobic compounds, while also conferring resistance to digestive enzymes, thereby enhancing their stability in the gastrointestinal tract [13]. Furthermore, studies have shown that PELNVs can accumulate at inflammatory sites via the enhanced permeability and retention (EPR) effect, and exhibit strong anti-inflammatory infiltration capabilities as well as immunomodulatory properties [14]. As a novel drug delivery system, PELNVs hold great potential to overcome the limitations of traditional herbal active compounds, such as poor solubility and low bioavailability.

In this study, we developed *Artemisiae Argyi*-derived exosome-like nanovesicles (AELNVs) and evaluated their therapeutic potential in alleviating LPS/D-GalN induced ALF in mice. Our research results show that oral administration of AELNVs alleviated hepatic pathological damage, significantly reduces the release of inflammatory mediators in serum, and increased survival rates in mice with ALF. Specifically, AELNVs suppress LPS/D-GalN-induced apoptotic signaling and NLRP3 inflammasome activation while inducing autophagy, thereby protecting hepatocytes from injury. Furthermore, AELNVs promote nuclear translocation of nuclear factor erythroid 2-related factor 2 (Nrf2), upregulate CYP2A5 expression, and mitigate oxidative stress and hepatotoxicity in ALF mice. Hepatic chemokine analysis revealed that AELNVs significantly downregulated CCL5/7 and CXCL9/10/11 expression, thereby reducing macrophage infiltration and alleviating hepatic inflammation. RNA sequencing combined with miRNA mimetic application identified MIR2916 as a key mediator through which AELNVs regulate the TLR4/NLRP3/Nrf2 signaling pathway to improve ALF prognosis. Collectively, this study highlights the therapeutic potential of AELNVs as a promising nanotherapeutic agent and provide novel strategies for treating acute liver failure.

## Materials and methods

### Extraction and purification of AELNVs

The isolation of AELNVs began with 1 kg of fresh *Artemisiae Argyi* foliage. The leaves were meticulously rinsed with phosphate-buffered saline (PBS) to eliminate surface contaminants, and subsequently homogenized using a high-performance blender. The crude homogenate was filtered through cheesecloth to remove particulate matter, yielding a clarified filtrate that was collected for further processing. Then the filtrate was subjected to three consecutive low-temperature high-speed centrifugations (4 °C, 10,000 g) for 60 min in order to eliminate plant residues, fibers, and other large particles, yielding a clear liquid as the crude extract. The crude extract was purified using a 0.45 μm PVDF membrane to remove low molecular weight protein impurities, and then loaded into the sample chamber of the Tangential Flow Filtration (TFF) system (Unique AutoTFF075, Inscinstech, Suzhou). Collect components smaller than 200 nm using a 200 nm membrane column, and continue to collect retained liquid (approximately 30 mL) of components larger than 750 kDa using a 750 kDa membrane column to obtain enriched and purified *Artemisia argyi* exosome like nanovesicles. Fresh *Artemisia argyi* were purchased from Qichun, Hubei Province, China, and were authenticated by Professor Yanfen Huang at Zhejiang Chinese Medical University.

### Characterization of AELNVs

The morphology of AELNVs was observed using transmission electron microscopy (TEM, HT 7700, Hitachi). The zeta potential and particle size of AELNVs were measured using resistive pulse sensing (RPS)(NanoCoulter counter, Resuntech, Shenzhen) and dynamic light scattering (DLS) (Zetasizer Nano ZS, Malvern Instruments, UK).

### In vitro digestion simulation of AELNVs

The digestibility of AELNVs was evaluated using an in vitro simulation model. Briefly, 100 μL of AELNVs was first subjected to an oral phase by mixing with 150 μL of artificial saliva (PHYGENE, PH1843) and incubating at 37 °C for 5 min. The mixture then entered the gastric phase with the addition of 300 μL of artificial gastric fluid (PHYGENE, PH1840), followed by incubation at 37 °C for 120 min. For the intestinal phase, 300 μL of artificial intestinal fluid (PHYGENE, PH1841) was introduced, and the incubation continued for 60 min at 37 °C. After the simulated digestion, the samples were centrifuged at 8,000 g. The changes in particle concentration and size of the digested products were determined by RPS.

### In vivo biodistribution

According to the reagent manual, incubate AELNVs with DIR dye at 37 °C for 30 min. Add an ultrafiltration tube and centrifuge at 8,000 g at 4 °C for 20 min to remove free dye, obtaining AELNVs-DIR, then resuspend in PBS. Administer a single dose of AELNVs-DIR per mouse via oral gavage to LPS/D-GalN-injured mice. Subsequently, the distribution of AELNVs-DIR in mice was detected at 0,1, 2, 4, and 6 h using an in vivo animal imaging system MOIS HT (RWD Life Science, Shenzhen, china).

### Animal experiments

Male BALB/c mice aged 6–8 weeks were used in this study. All mice are housed in pathogen free (SPF) facilities and have free access to food and water. The housing conditions are set to a 12 h/12 h light dark cycle, with a room temperature of 25° C and a relative humidity of 50–60%. All experimental protocols have been approved by the Ethics Committee of Zhejiang Provincial People's Hospital (Approval Number: 20250902654658).

To establish an ALF model, mice underwent at least one week of adaptation period under these housing conditions and received intraperitoneal injections of LPS (100 μg/kg) and D-GalN (700 mg/kg). One hour after induction, the mice in the treatment group were orally administered 100 mg/kg of AELNV as a treatment method, while the control group animals received the same volume of PBS. Then collect serum and liver tissue samples for further analysis. Upon euthanasia, which was performed 6 h after the LPS/D-GalN challenge, blood and liver tissue specimens were collected for further processing. Survival rate monitoring terminated within 24 h.

### Histological analysis

Fresh mouse liver tissues were fixed in 4% paraformaldehyde, paraffin-embedded, and sectioned at 5 μm for histological analysis. For overall morphology, sections were stained with hematoxylin and eosin (H&E) and examined by light microscopy. For immunohistochemistry, dewaxed and rehydrated sections were blocked with goat serum, sequentially incubated with primary and secondary antibodies, followed by avidin–biotin-peroxidase complex, and developed with diaminobenzidine (DAB); results were quantified using ImageJ. For immunofluorescence detection, the tissue sections were blocked with 1.5% goat serum to reduce nonspecific binding, followed by overnight incubation at 4 °C with the primary antibody, and subsequently incubated with a fluorophore-conjugated secondary antibody. Signals were visualized by laser confocal microscopy and analyzed for area and intensity with ImageJ.

### RT-qPCR

Total RNA was extracted from liver tissue or cultured cells using TRIzol reagent, followed by reverse transcription into cDNA using a cDNA synthesis kit (Novoprotein, catalog number E147). Real-time PCR amplification was monitored using SYBR Green qPCR SuperMix (Novoprotein, catalog number E096) on an Applied Biosystems 7500 Real-Time PCR System (Thermo Scientific). Gene expression levels were normalized to the housekeeping gene Gapdh and calculated using the 2^(-ΔΔCt) method. Gene-specific primers were synthesized by Sangon (Shanghai, China), and their sequences are provided in Table 1 of the Supplementary Materials.

### Western blot

Liver tissue or cultured cells were lysed using RIPA buffer supplemented with protease inhibitors. Protein concentration was determined by BCA assay, and equal amounts of protein were separated by SDS-PAGE followed by transfer onto PVDF membranes. The membranes were blocked with 5% skim milk at room temperature for 1 h and then incubated with primary antibody overnight at 4 °C. After washing with TBST, the membranes were incubated with HRP-conjugated secondary antibody at room temperature for 1 h. Protein bands were visualized using enhanced chemiluminescence (ECL) substrates and captured using a chemiluminescence imaging system. Densitometric quantification was performed using ImageJ software. Detailed information for all antibodies used in the Western blot experiments is provided in Table 2 of the Supplementary Materials.

### Biochemical assay

Following euthanasia, blood samples were collected from all mice and centrifuged to obtain serum. Serum levels of Alanine Aminotransferase (ALT), Aspartate Aminotransferase (AST), Lactate Dehydrogenase (LDH) were measured using an automated biochemical analyzer. Concurrently, liver tissue homogenates were prepared to assess the levels of Glutathione (GSH), Glutathione Peroxidase (GPx), Myeloperoxidase (MPO) and Malondialdehyde (MDA) using specific commercial assay kits (Nanjing Jiancheng Bioengineering Institute, China).

### ELISA

The concentrations of TNF-α, IL-1β, IL-6, IFN-γ, IL-4, and IL-10 in liver tissue were quantified using a specific anti-mouse ELISA kit according to the manufacturer’s instructions (Jianglai Biotechnology, China). The optical density was measured at 450 nm, and cytokine concentrations were determined by interpolation from the standard curve.

### Coumarin 7'-hydroxylase assay

According to the manufacturer's instructions, liver microsomes were prepared from frozen liver tissue using the BioVision Microsomal isolation Kit (Cat.No.1175015, K249-50). The CYP2A5 enzyme activity was detected as mentioned earlier [15]. The activity of coumarin 7'-hydroxylase was calculated based on the pmol/min/mg microsomal protein of the formed 7'-hydroxycoumarin.

### Dual-luciferase reporter assay

The dual luciferase reporter assay was used to verify whether TLR4 is a direct target gene of miR-2916. Specifically, in the pmirGLO vector, the TLR4 3'- UTR fragment containing the predicted wild-type (WT) miR-2916 binding site was cloned downstream of the firefly luciferase gene. Generate mutant (MUT) constructs carrying mutations in the seed sequence as controls. WT or MUT reporter plasmids and miR-2916 mimetics or negative control miRNA (miR-NC) were co transfected into HEK-293T cells. Measure the luciferase activity in cells 48 h after transfection using a dual luciferase reporter kit (Vazyme, DL101-01, China)). The firefly luciferase activity was standardized to Renilla luciferase activity for each sample.

### Isolation of liver immune cells

Mice were perfused with PBS through the portal vein, followed by infusion with collagenase IV solution. Then mechanically separate and digest the excised liver. Pass the obtained cell suspension through a 70 μ m cell filter. Remove liver cells by centrifugation using a 30–40% Percoll density gradient. The obtained non parenchymal cell portion will be subjected to red blood cell lysis, washing, and resuspended in buffer to obtain liver immune cells for subsequent flow cytometry analysis.

### Liver RNA-seq

Following RNA quality assessment using the Agilent 2100 Bioanalyzer, a sequencing library was constructed. Briefly, mRNA was enriched and fragmented via poly(A) selection. In the reverse transcriptase system, fragmented AELNV mRNA is used as a template and random oligonucleotide primers for first strand cDNA synthesis. Subsequently, the double stranded cDNA was purified by synthesizing the second strand cDNA using DNA polymerase I, followed by terminal repair and A-tailing, and connected to sequencing adapter. Fragments of 370–420 bp were selected using AMPure XP beads and the final library quality was evaluated using a Qubit fluorometer, the Agilent 2100 Bioanalyzer, and RT-qPCR to ensure accurate quantification prior to sequencing. Perform functional enrichment analysis on differentially expressed genes using the ClusterProfiler software package.

### miRNA-sequencing

Sequencing libraries for miRNA were constructed from total RNA of AELNVs by size-selecting small RNAs (18–30 nt), followed by 3' and 5' adapter ligation, reverse transcription, and PCR amplification. The purified libraries were sequenced on an Illumina HiSeq Xten platform. The raw data were processed through a bioinformatics pipeline that included adapter trimming and quality control. High-quality reads were sequentially aligned to the Rfam (v11.0) and GenBank (v209.0) databases for annotation. Target genes of identified miRNAs were predicted using and TargetScan (v7.0), followed by functional enrichment analysis of these targets via GO terms and KEGG pathways.

### Statistical analysis

Statistical analyses were performed by GraphPad Prism 10.1. Data from multiple groups were analyzed by one-way ANOVA with Dunnett’s T3 test, and two-group comparisons were made using t-tests. All quantitative data are reported as mean ± SEM, with statistical significance defined as *p < 0.05, **p < 0.01, ***p < 0.001, and ****p < 0.0001.

## Results

### Characterization of AELNVs

**Fig. 1:**
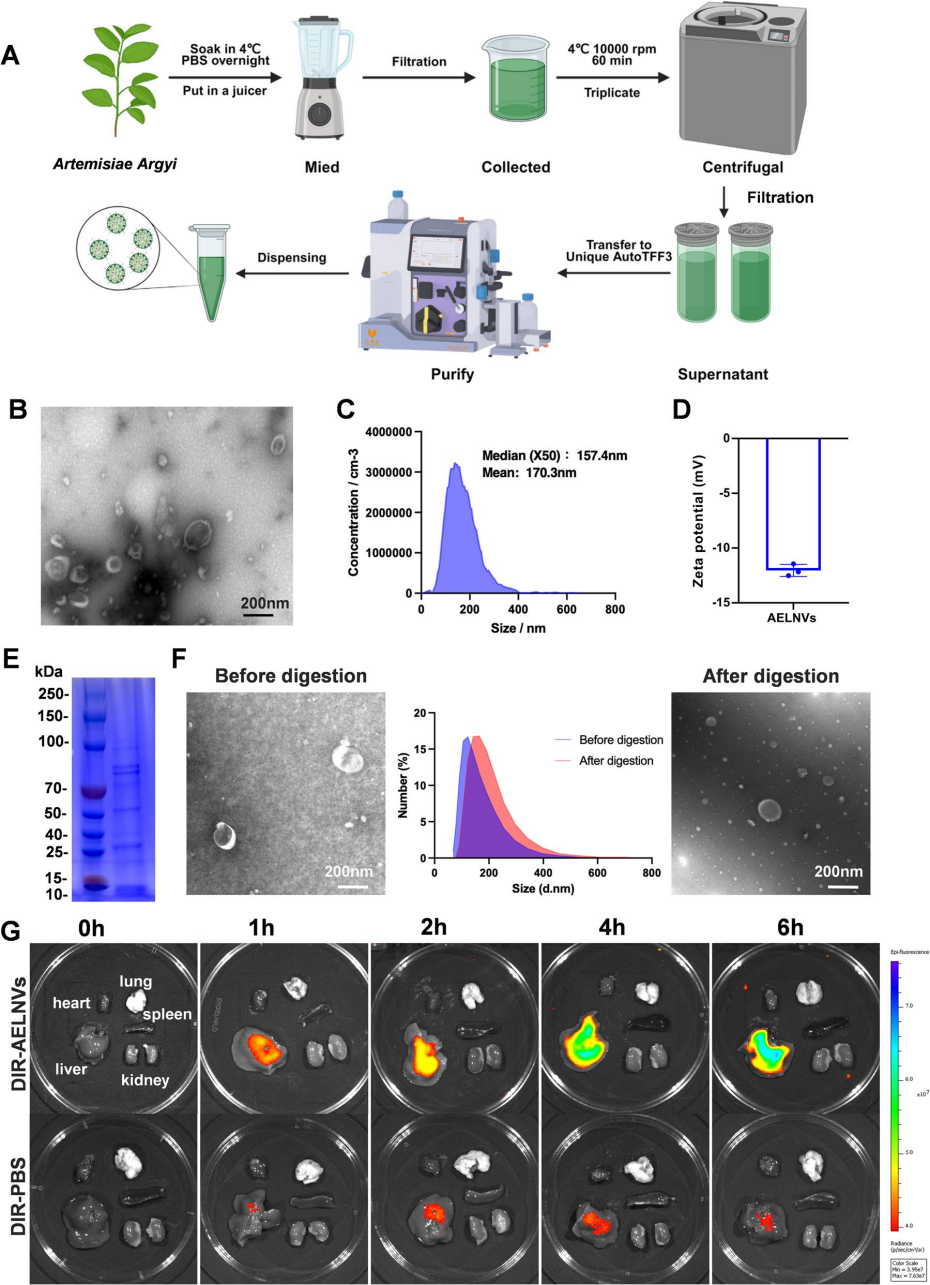
Identification and characterization of AELNVs. **A** The schematic illustration of the isolation and purification process of AELNVs. **B** Characterization of the morphology and size of AELNVs by transmission electron microscopy (TEM). Scale bar: 200 nm. **C** The size of AELNVs was characterized by RPS analysis. **D** The surface zeta potential of the AELNVs was detected in Malvern Nano ZS. **E** Protein gel by coomassie brilliant blue staining. **F** Particle size distribution and TEM images of AELNVs before and after simulated digestion. **G** Distribution of DIR-labeled AELNVs and the same amount of DIR without AELNVs in main organs of mice at different time points

To purify AELNVs, fresh *Artemisiae Argyi* leaves were processed into juice and filtered and centrifuged at high speed to remove plant residues, fibers, and other large particles, resulting in a crude extract. Enriched and purified *Artemisiae Argyi* exosome nanovesicles were obtained using Tangential Flow Filtration (TFF) (Fig. 1A). Transmission electron microscopy (TEM) observation revealed that AELNVs have characteristic circular or cup-shaped morphologies and disc-shaped concave structures (Fig. 1B). The particle size distribution range of AELNVs measured using a NanoCoulter counter is approximately 95.7–248.3 nm, and the surface Zeta potential is approximately −11.82 mV (Fig. 1C, D). SDS-PAGE showed that the protein content of AELNVs was within the molecular weight range of 10–250 kDa (Fig. 1E). BIO-Fragment Analyzer showed that most of the RNAs in AELNVs were composed of small RNA species (Figure S1).

Furthermore, we evaluated the stability of AELNV in simulated gastrointestinal fluid to assess its suitability for oral administration. The results showed that the morphological characteristics of AELNV remained almost unchanged in gastric and intestinal like environments, with a slight increase in particle size. This stability indicates that AELNVs are suitable for oral administration (Fig. 1F, Figure S2). Meanwhile, we investigated the in vivo distribution of orally administered AELNVs. Mice with LPS/D-GalN injury received DIR labeled AELNVs (100 mg/kg), and their fluorescence intensities were monitored using imaging techniques at 0, 1, 2, 4, and 6 h. Within 6 h after administration, the fluorescence intensity in the liver of mice gradually increased over time, indicating that AELNVs were effectively enriched in the damaged liver (Fig. 1G).

### AELNVs exert a protective effect against LPS/D-GalN-induced ALF

**Fig. 2:**
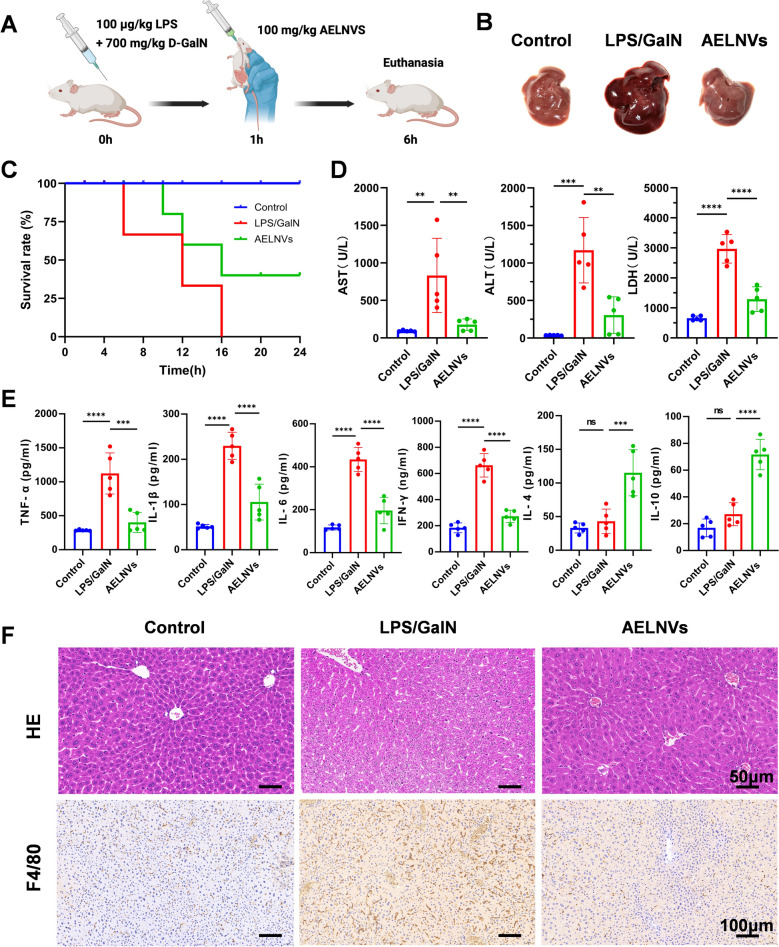
AELNVs alleviate LPS/D-GalN-induced hepatotoxicity and pathological injury. **A** Schematic diagram of ALF mouse model establishment and treatment protocol. **B** The representative image of the liver demonstrates the therapeutic effect of AELNVs. **C** Mouse survival rates (%) **D** ALT, LDH and AST levels in serum 6 h after LPS/D-GalN treatment (n = 5 per group). Data represent means ± SEM. Statistical analysis was performed by one-way ANOVA. *p < 0.05, **p < 0.01, ***p < 0.001, ****p < 0.0001. **E** The expression of inflammatory cytokines TNF-α, IL-1β, IL-6, IFN-γ, IL-4 and IL-10 in liver protein was detected by ELISA (n = 5, per group). Data represent means ± SEM. Statistical analysis was performed by one-way ANOVA. *p < 0.05, **p < 0.01, ***p < 0.001, ****p < 0.0001. ns, no significance. **F** Pathological changes in liver tissue are presented with H&E staining (Scale bar: 50 μm), and representative immunohistochemical staining plots for F4/80 (Scale bar: 100 μm)

In order to investigate the therapeutic effect of AELNVs on acute liver failure, intervention studies were conducted in a mouse model induced by LPS/D-GalN. As shown in Fig. 2A, AELNVs (100 mg/kg) were orally administered 1 h after induction. The results revealed that AELNVs treatment conferred significant protection against LPS/D-GalN-induced liver injury, as evidenced by a marked improvement in survival rates (Fig. 2B, C). In terms of hepatic injury biomarkers, mice subjected to acute liver injury exhibited substantial increases in serum levels of ALT, AST, and LDH. However, administration of AELNVs notably suppressed the LPS/D-GalN-induced elevation of these enzymes, indicating stabilization of hepatocyte membranes and attenuation of hepatocellular necrosis (Fig. 2D).

Under the influence of LPS/D-GalN, hepatic macrophages become activated and release substantial quantities of pro-inflammatory mediators. This triggering event elicits pronounced hepatocellular mortality, thereby aggravating the ensuing inflammatory cascade [2]. ELISA analysis (Fig. 2E) demonstrated that AELNVs not only significantly suppressed the production of key pro-inflammatory factors TNF-α, IL-1β, IL-6, and IFN-γ in the livers of ALF mice, but also promoted the expression of anti-inflammatory factors IL-4 and IL-10. Administration of AELNVs effectively regulated the inflammatory response. Histopathology and immunohistochemical analysis similarly revealed the livers of AELNVs therapy markedly improved structure, characterized by uniform hepatocyte arrangement, intact hepatic lobules and significant reduction in hepatic macrophages (Fig. 2F). In conclusion, AELNVs exert a hepatoprotective effect, effectively suppressing inflammatory responses and liver injury in LPS/D-GalN-treated mice.

**Fig. 3:**
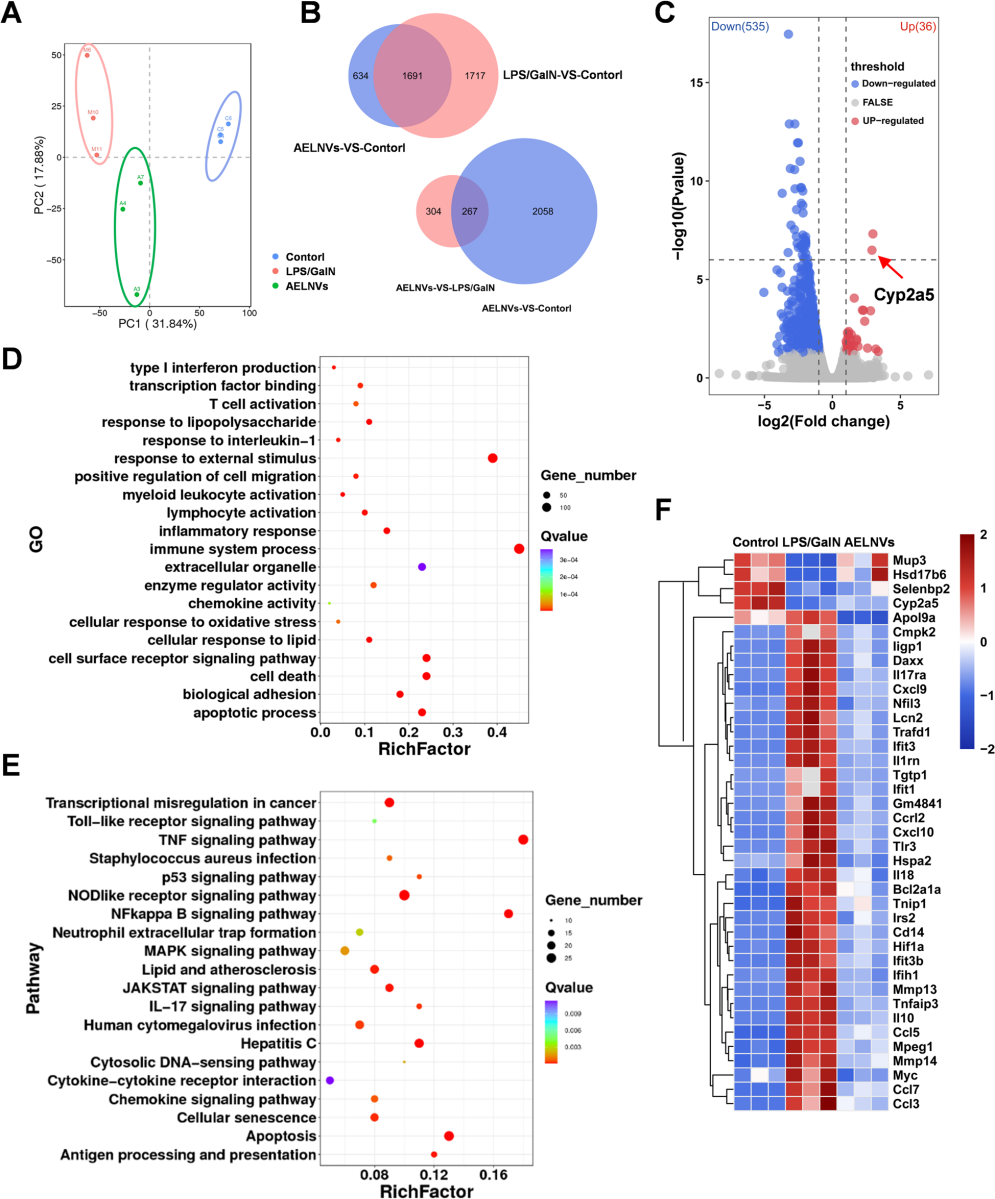
AELNVs alleviate acute liver failure: RNA-seq analysis. **A** Principal Component Analysis (PCA) **B** Venn plot displays the distribution patterns and differential gene expression analysis among the three groups. **C** The volcano plot represents a significant decrease or increase in gene expression compared to the LPS/D-GalN model group, as indicated by the green and red dots in the AELNVs treatment group (p < 0.05). The x-axis represents the expression of log2FoldChange, and the y-axis represents the significance level of gene expression differences [−log10 (p < 0.05)]. **D** The significant GO terms were selected and the Dotplot was employed. The x-axis showed the ratio of the number of differential genes annotated to the GO terms to the total number of differential genes. The y-axis represented the GO terms. **E** The significant 20 KEGG pathways were selected and the Dotplot was employed. The x-axis showed the ratio of the number of differential genes annotated to the KEGG pathways to the total number of differential genes. The y-axis represented the KEGG pathways. **F** The heatmap results of RNA-Seq analysis revealed the key genes whose expressions exhibited significant alterations among the three groups. The size of the dot represented the number of genes annotated to KEGG pathways/GO terms, and the color from red to bule represents the significance of the enrichment (p < 0.05)

### AELNVs alleviate acute liver failure: RNA-seq

To understand how oral administration of AELNVs alleviates ALF in mice, we used RNA-seq to investigate the potential protective mechanism of AELNVs in ALF. Three groups of nine liver specimens were sequenced: control group, LPS/D-GalN model group, and AELNVs treatment group. Principal Component Analysis (PCA) showed significant differences in the distribution patterns among the three groups (Fig. 3A). The Venn plot (Fig. 3B) and volcano plot (Fig. 3C) visually demonstrated the significant differences in gene expression in the liver of mice receiving AELNVs prevention and treatment compared to the LPS/D-GalN model. Specifically, treatment with AELNVs significantly upregulated the expression levels of 36 genes. Meanwhile, the expression of p53 genes was significantly downregulated.

Gene Ontology (GO) analysis (Fig. 3D) showed that treatment with AELNVs significantly affected the top 20 metabolic processes, including transcription factor binding, immune system processes, T cell activation, cellular response to oxidative stress, and cell death, etc. KEGG (Kyoto Encyclopedia of Genes and Genomes) enrichment analysis (Fig. 3E) identified 20 signaling pathways that were most enriched in the AELNVs treatment group, mainly including TNF signaling pathway, Chemokine signaling pathway, NF-κB signaling pathway, Apoptosis, etc. The heatmap results of RNA-Seq analysis (Fig. 3F) demonstrated that AELNVs significantly modulated the expression levels of key genes associated with pathways including oxidative stress, apoptosis, and inflammatory cytokines. This finding implied that AELNVs may mitigate the progression of acute liver failure by regulating the aforementioned signaling pathways.

### AELNVs prevent hepatocyte apoptosis by inhibiting the TLR4/NF-κB/NLRP3 signaling axis in ALF mice

**Fig. 4:**
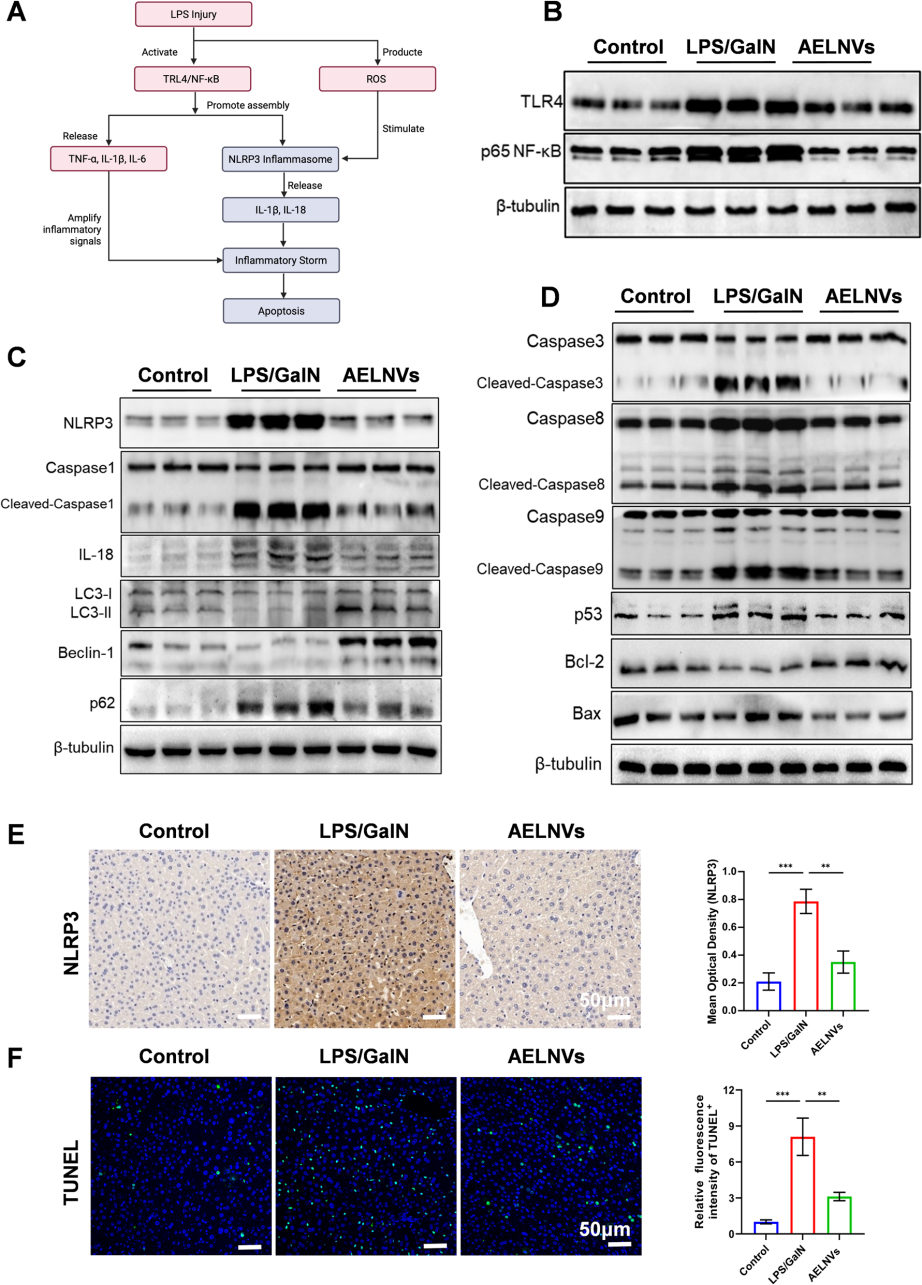
AELNVs prevent hepatocyte apoptosis by inhibiting the TLR4/NF-κB/NLRP3 signaling axis in ALF mice. **A** Schematic diagram of LPS triggering an inflammatory cascade and apoptosis by activating the TLR4/NF-κB pathway. **B** Protein expression of TLR4 and NF-κB P65 in liver tissues were analyzed by western blot (n = 3 per group). **C** Western blot analysis of NLRP3, Caspase1, IL18, LC3-I, LC3-II, Beclin-1and p62 protein activation and expression in liver tissue (n = 3 per group). **D** Mouse liver tissue collected 6 h after LPS/GalN challenge. Western blot analysis of AELNV effects on apoptosis-related proteins Caspase3, Caspase8, Caspase9, p53, Bcl2 and Bax expression (n = 3 per group). **E** Immunohistochemical analysis of NLRP3 in liver tissue (n = 3 per group). Scale bar: 50 μm. **F** TUNEL assay evaluating hepatic apoptosis in mice (left) and quantitative results (right) (n = 3 per group). Scale bar: 50 μm. Data are presented as mean ± standard error. Statistical analysis was performed using one-way ANOVA. *p < 0.05, **p < 0.01, ***p < 0.001, ****p < 0.0001, ns indicates no statistical significance

LPS activates the TLR4/NF-κB pathway in macrophages, triggering a cascade of inflammatory responses and a “cytokine storm” that promotes widespread hepatocyte apoptosis, a key driver of ALF (Fig. 4A) [1, 3, 16]. Our results showed that AELNVs treatment effectively suppressed the LPS/D-GalN-induced upregulation of TLR4 and subsequent NF-κB activation (Fig. 4B, Figure S3). Furthermore, AELNVs inhibited the NLRP3 inflammasome pathway, reflected in reduced levels of NLRP3, cleaved Caspase-1, and IL-18, and concurrently promoted autophagy, as indicated by elevated Beclin-1 and an increased LC3-II/LC3-I ratio (Fig. 4C, Figure S4). These suggested that AELNVs mitigate inflammation partly by promoting autophagy, which negatively regulates NLRP3 activation [17]. In addition, AELNVs exerted strong anti-apoptotic effects. They significantly downregulated the expression of pro-apoptotic mediators, including p53, cleaved Caspase-9, -8, and -3, and concomitantly increased the Bcl-2/Bax ratio in the injured liver tissue. (Fig. 4D, Figure S5). Immunohistochemistry confirmed the inhibition of NLRP3 and IL-1β activation (Fig. 4E, Figure S6), and TUNEL staining revealed a marked reduction in apoptotic cells following AELNVs treatment (Fig. 4F).

### AELNVs alleviate oxidative stress in ALF via the Nrf2-CYP2A5 signaling pathway

**Fig. 5:**
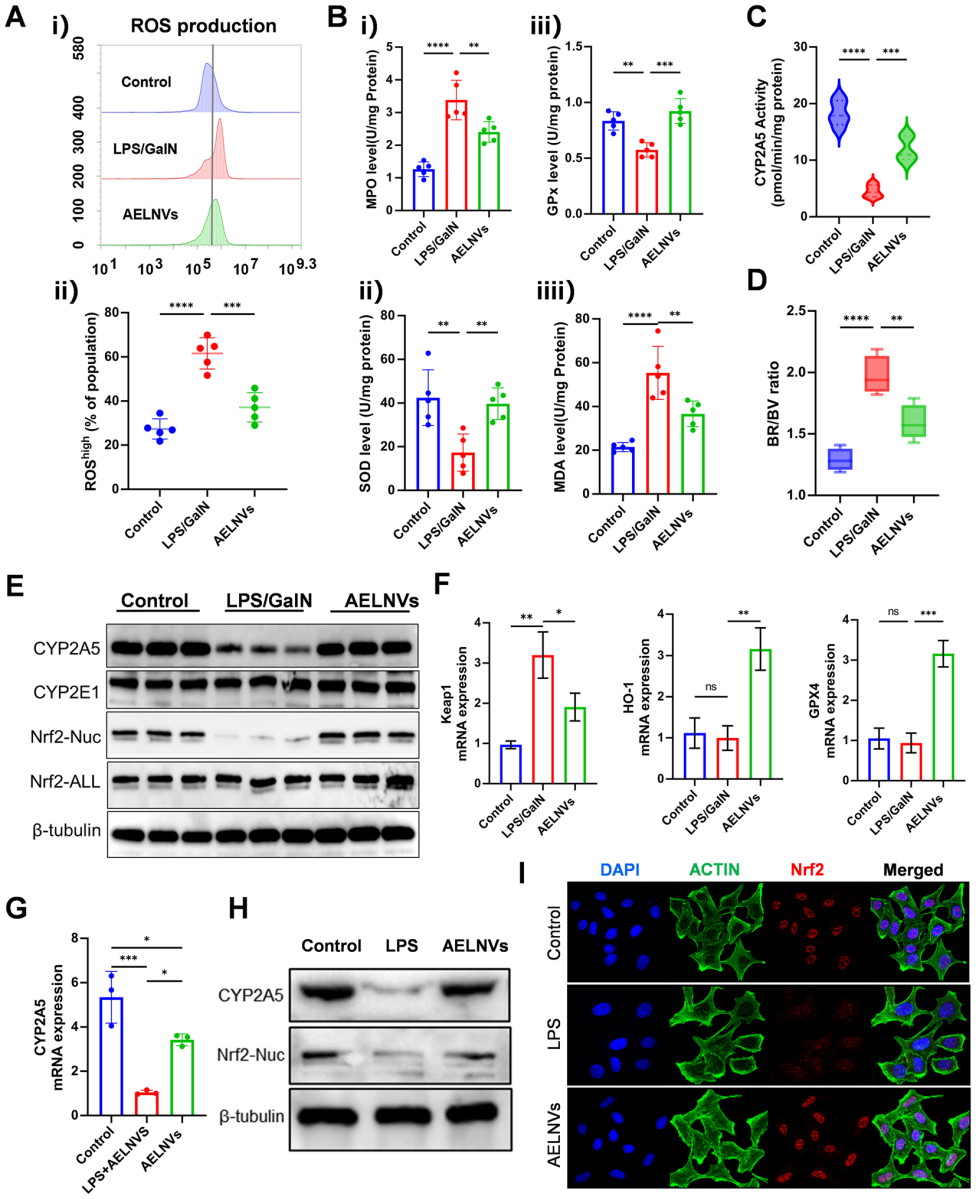
AELNVs alleviate oxidative stress in ALF via the Nrf2-CYP2A5 signaling pathway. **A** Flow cytometry detection of reactive ROS production. **B** Effects of AELNVs on GSH, MPO, MDA, and GPx enzyme activity. (n = 5 per group). **C** CYP2A5 enzyme activity is detected by measuring coumarin 7 '- hydroxylase. **D** The ratio of bilirubin (BR) to biliverdin (BV) is detected using the corresponding biochemical reagent kit. **E** Protein expression of Cyp2A5, Cyp2E1, Nrf2 (Nuclear proteins and all proteins) in liver tissues were analyzed by western blot (n = 3 per group). **F** Expression of Kelch like ECH related protein (Keap1), heme oxygenase-1 (HO-1), glutathione peroxidase 4 (GPX4) in mouse liver tissues mRNA (n = 5 per group). **G** The effect of AELNVs on the mRNA levels of Cyp2A5 was detected by qPCR. Huh7 cells were pretreated with LPS (1 mg/ml) for 6 h. Then, AELNVs (1 μg/ml) was added and incubated for 12 h, then cells were collected for RT-qPCR assay. **H** Protein expression of Cyp2A5, Nrf2 (Nuclear proteins and all proteins) in Huh7 cells were analyzed by western blot (n = 3 per group). **I** Detection of Nrf2 protein nuclear localization in Huh7 cells by immunofluorescence staining. Data represent means ± SEM. Statistical analysis was performed by one-way ANOVA. *p < 0.05, **p < 0.01, ***p < 0.001, ****p < 0.0001. ns, no significance

Oxidative stress plays a pivotal role in LPS/D-GalN-induced hepatotoxicity. We first measured levels of reactive oxygen species (ROS), reduced glutathione (GSH), glutathione peroxidase (GPx), myeloperoxidase (MPO), and malondialdehyde (MDA) levels in mouse liver tissue. We found that AELNVs effectively alleviated the LPS/D-GalN-induced elevation of oxidative stress (Fig. 5A, B). RNA sequencing analysis revealed that oral administration of AELNVs significantly enhanced cytochrome P450 2A5 (CYP2A5) gene expression in ALF mice (Fig. 3C). CYP2A5 plays a crucial role in heme and bilirubin (BR) metabolism. High bilirubin concentrations generate reactive oxygen species and induce hepatocyte apoptosis; this enzyme oxidizes toxic bilirubin into less harmful biliverdin (BV) [18]. We found that AELNVs restored CYP2A5 enzyme activity (Fig. 5C), thereby reducing the BR/BV ratio (Fig. 5D) in ALF mice. Western blotting results also validated the alleviation of CYP2A5 expression suppression in ALF mice by AELNVs (Fig. 5E). The expression of CYP2A5 is transcriptionally regulated by Nrf2. When oxidative stress occurs, Nrf2 separates from the cytoplasmic inhibitor Keap1, transfers to the nucleus, and activates antioxidant genes including CYP2A5 and heme oxygenase-1 (HO-1) [19]. In AELNVs-treated ALF mice, although total Nrf2 protein levels remained unchanged, its nuclear accumulation was significantly enhanced (Fig. 5E), accompanied by Keap1 downregulation and upregulation of HO-1 and GPX4 (Fig. 5F). Consistent with this, AELNVs similarly upregulated CYP2A5 and promoted Nrf2 nuclear translocation in Huh7 cells in vitro (Fig. 5G–I). These findings indicate that AELNVs mitigate oxidative liver injury by regulating the Nrf2/CYP2A5 pathway.

### AELNVs alleviate liver immune response in ALF mice by inhibiting chemokines

**Fig. 6:**
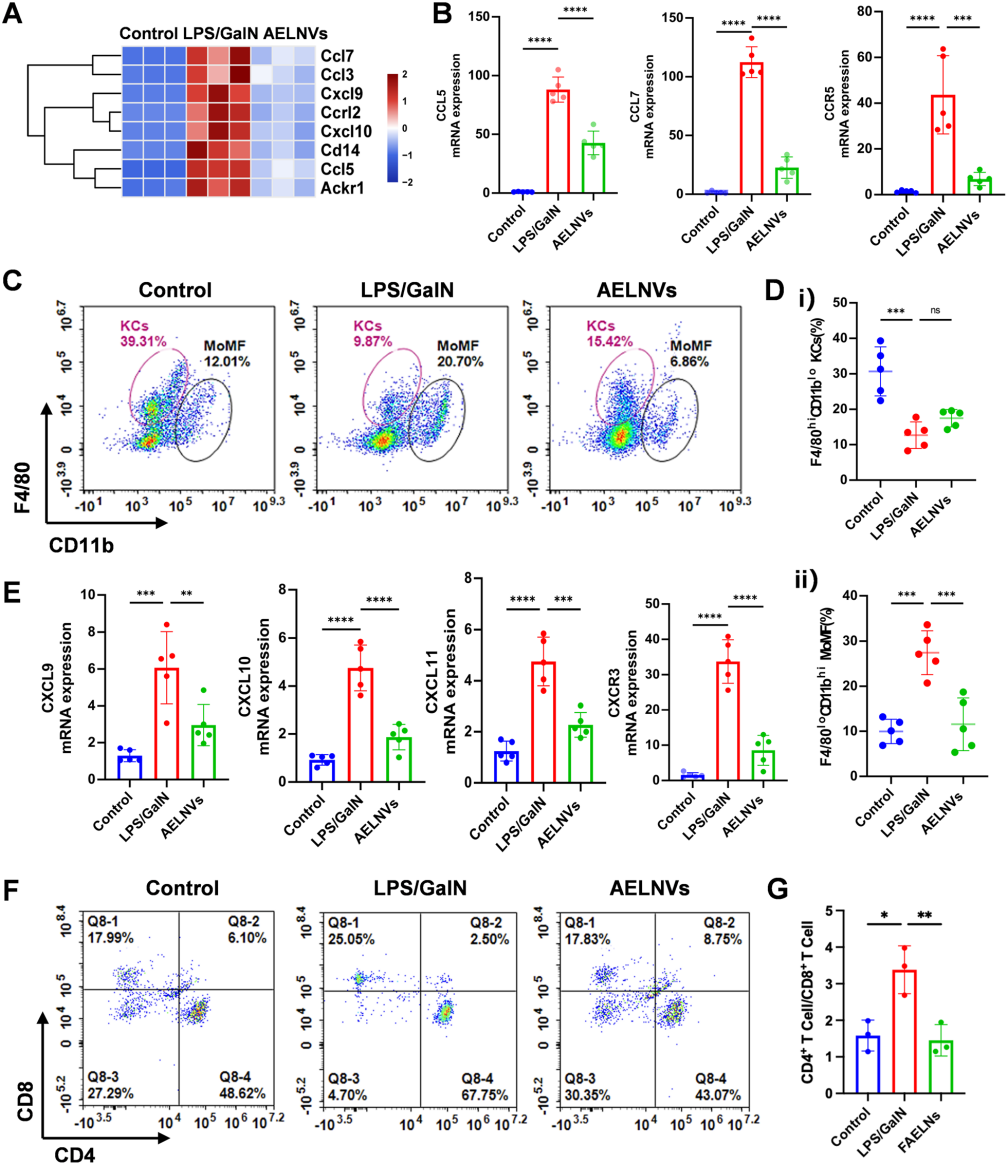
AELNVs alleviate ALF by inhibiting CCL5/7-CCR5 and CXCL9/10/11-CXCR3 axes in the liver of ALF mice. **A** The heatmap of chemokines in RNA-Seq analysis. **B** Expression of chemokines CCL5, CCL7 and CCR5 in mouse liver mRNA (n = 5 per group). **C**, **D** Analyzing and quantificating of F4/80^hi^CD11b^lo^ resident Kupffer cells, and F4/80^lo^CD11b^hi^ infiltrating monocyte-derived hepatic macrophages using flow cytometry (n = 5 per group). **E** Expression of chemokines CXCL9/10/11 and CXCR3 in mouse liver mRNA (n = 5 per group). **F**, **G** Analyzing and quantificating CD4^+^ T cells and CD8.^+^ T cells in liver by flow cytometry (n = 5 per group). Data represent means ± SEM. Statistical analysis was performed by one-way ANOVA. *p < 0.05, **p < 0.01, ***p < 0.001, ****p < 0.0001

RNA-seq analysis revealed that oral administration of AELNVs affected the chemokine signaling pathway in ALF mice, with AELNVs downregulating chemokines including CCL5, CCL7, CXCL9, and CXCL10 in ALF mice (Fig. 6A). We verified these chemokines with significantly changed expression levels in the heatmap by RT-qPCR. The chemokines CCL5/7 interact with their receptor CCR5 (C-chemokine receptor 5), participating in immune cell migration, signaling, and inflammation, thereby promoting the migration of monocytes and macrophages to sites of inflammation and tissue injury [20]. LPS/D-GalN stimulation upregulated the hepatic expression of chemokines CCL5/7 and their receptor CCR5, which was notably suppressed by AELNVs administration, indicating a potential reduction in monocyte recruitment. (Fig. 6B). Therefore, flow cytometric analysis of hepatic mononuclear cells was performed to evaluate the infiltration of circulating monocyte-derived macrophages (MoMFs) in response to LPS/D-GalN-induced liver injury. Results (Fig. 6C, D) showed that, compared with the injury group, the AELNVs treatment group exhibited a slight increase in the number of liver-resident Kupffer cells (F4/80^hi^CD11b^lo^), though this did not reach statistical significance. However, AELNVs treatment significantly reduced the abundance of recruited F4/80^lo^CD11^hi^-MoMF cells recruited. This suggested that AELNVs alleviate liver injury by inhibiting the chemokine signaling axis, thereby decreasing the recruitment of inflammatory monocyte-macrophages to the liver.

We also evaluated the effect of AELNVs intervention on the polarization of hepatic macrophages. Immunophenotyping demonstrated that LPS/GalN challenge enriched pro-inflammatory F4/80⁺CD86⁺ M1 macrophages in the liver. Treatment with AELNVs effectively redistributed this population and augmenting the frequency of F4/80⁺CD206⁺ M2 macrophages (Figure S7). This shift aligns with established evidence that autophagy activation in macrophages inhibits the M1 phenotype and promotes M2 polarization [21], suggesting a mechanism through which AELNVs exert their immunomodulatory function. Meanwhile, AELNVs treatment inhibited the CXCL9/10/11-CXCR3 (Fig. 6E) axis and lowered the hepatic CD4^+^/CD8^+^ T cell ratio (Fig. 6F, G), which collectively contributed to the modulation of the inflammatory response. These results demonstrated that AELNVs prevent the hepatic infiltration of inflammatory monocytes in response to exogenous toxin-induced injury.

### The miRNA profiling of AELNVs

**Fig. 7:**
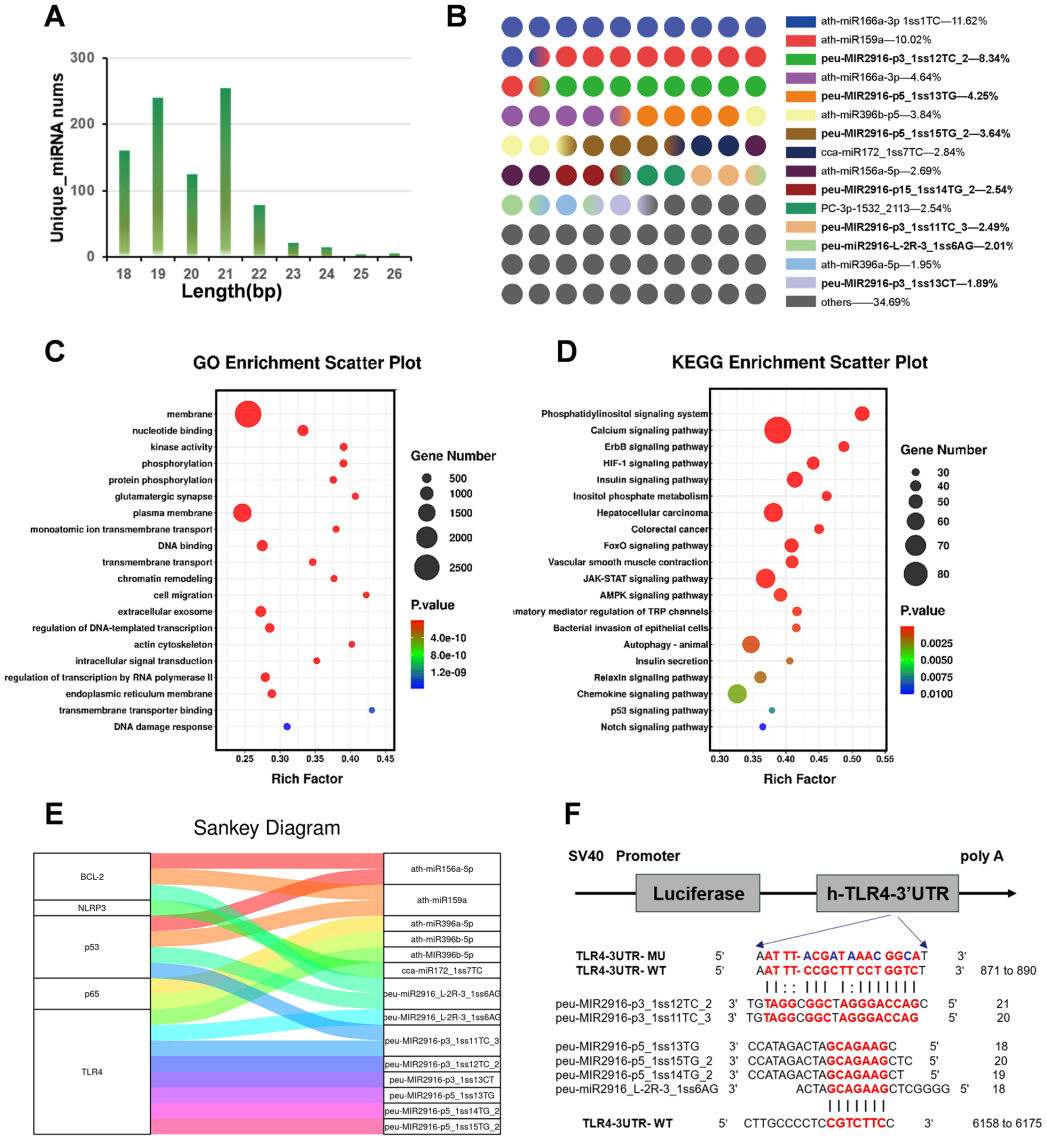
The miRNA profiling of AELNVs. **A** Length distribution of clean reads of AELNVs small RNA libraries. **B** The top 15 miRNAs with the highest expression levels of AELNVs were displayed. **C** Gene Ontology (GO) enrichment analysis (biological processes) of target miRNA genes in AELNVs. **D** KEGG enrichment analysis based on the target genes of miRNA in AELNVs. The size of the dot represented the number of genes annotated to KEGG pathways/GO terms, and the color from red to bule represents the significance of the enrichment (p < 0.05). **E** Sankey plot shows the association between the top 15% of miRNAs expressed in AELNVs and target genes NLRP3, NF-κB P65 (RelA), TNFR, TLR4, and Keap1 predicted using targeted scanning (version 7.0) software. **F** The schematic diagram shows the predicted double stranded structure formed between the 3'UTR of the human TLR4 gene and the miRNA (red) of the peu-MIR2916-like sequence, and exhibits two different base pairing structures. The mutated nucleotides of the TLR4-MU plasmid are shown in blue. Data represent means ± SEM. Statistical analysis was performed by one-way ANOVA. *p < 0.05, **p < 0.01, ***p < 0.001, ****p < 0.0001

MicroRNA (miRNA) is an important functional component of EVs. Recent studies have found that PELNVs contain various miRNAs, which can serve as biologically active molecular mediators to participate in cross species signal communication between medicinal plants and humans, and exert biological effects by regulating target gene expression [22, 23]. To investigate the role of miRNAs in alleviating acute liver failure in mice through AELNVs, we extracted miRNAs from AELNV samples and conducted deep sequencing. Following quality control and read filtering, we identified 902 unique miRNA sequences along with their corresponding copy numbers. We analyzed the lengths of the identified miRNAs; the abscissa represents the length of each miRNA, while the ordinate indicates the number of unique miRNAs after removing duplicates (the rationale for duplicate removal stems from simultaneous alignment of a miRNA precursor to both genomic positions). Notably, over 95% of these miRNAs ranged between 18 and 26 nucleotides in length (Fig. 7A). Interestingly, by comparing with existing miRNA databases, we discovered several peu-MIR 2916-like sequences among the top 15% of expressed miRNAs in AELNVs. such as peu-MIR2916-p3_1ss12TC2, peu-MIR2916-p5_1ss13TG, peu-MIR2916-p5_1ss15TG2, etc. (Fig. 7B). Subsequently, Gene Ontology (GO) enrichment analysis was performed on the target genes of miRNAs with a read count greater than 0.5% of the total sample content. The results indicated that these target genes were primarily involved in nucleotide binding, kinase activity, gene expression regulation within endoplasmic reticulum membranes, and other cellular metabolic processes (Fig. 7C). Furthermore, KEGG pathway analysis revealed that the top twenty metabolic pathways influenced by these target genes included calcium signaling pathway, HIF-1 signaling pathway, insulin signaling pathway as well as autophagy-animal etc. (Fig. 7D). Collectively, these findings suggest that miRNAs enriched in plant extracellular vesicle-like vesicles have potential biological roles in regulating cell metabolism across species.

The above studies indicate that AELNVs exert protective effects against liver injury by activating autophagy, thereby inhibiting oxidative stress, suppressing inflammasome activation and hepatocyte apoptosis, and reducing the release of inflammatory cytokines. We further investigated whether this inhibition is mediated by miRNAs derived from AELNVs. Five genes were selected for analysis, including NLRP3 and its upstream signaling components—NF-κB p65, TLR4, and p53—as well as BCL-2. were selected as putative target genes. Using the Targeted Scan (Version 7.0) software, we predicted the binding of miRNAs in AELNVs to these putative target genes. The results of the Sankey plot (Fig. 7E) and Gene regulatory network diagram (Figure S8) indicate that the six miRNAs with peu-MIR 2916 like sequences in the top 15% of miRNAs expressed in AELNV are significantly correlated with the TLR4 gene. According to the prediction results of Miranda software (version 3.3a), the miRNAs of these six peu MIR 2916-like sequences all have strong binding with the 3'UTR sequence of TLR4, and exhibit two different base-pairing structures, with the minimum free energy (MFE) of -9.74 kCal/Mol (Fig. 7F). TLR4 plays a crucial role in mediating organ damage in ALF, and inhibition of TLR4 signaling has been shown to ameliorate liver dysfunction, representing a potential therapeutic strategy [24]. These data suggest that miRNAs in AELNVs may exert hepatoprotective effects by inhibiting the gene expression of TLR4 in liver tissues of ALF mice.

### AELNVs-derived peu-MIR2916 can downregulate TLR4 gene expression

**Fig. 8:**
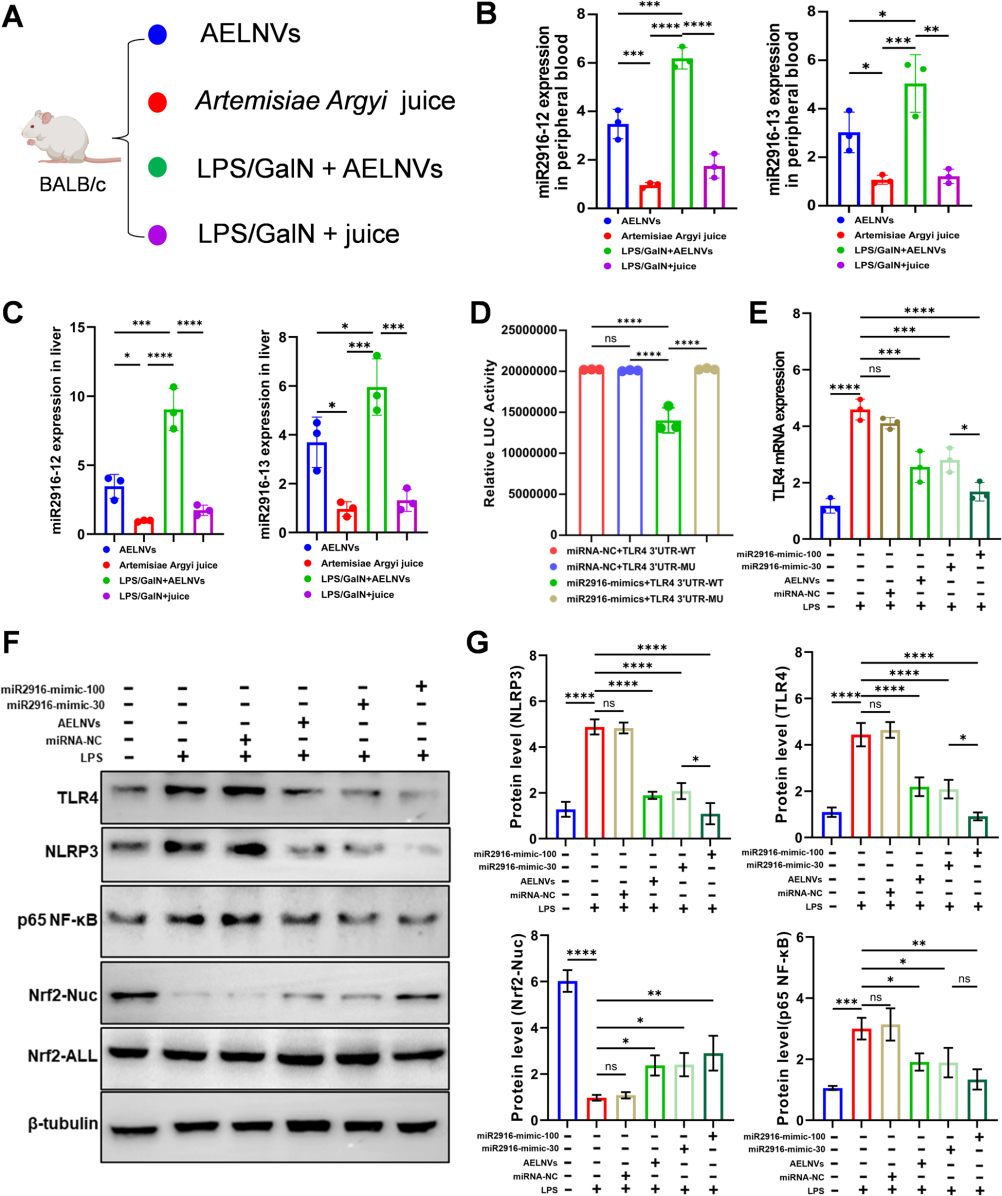
AELNVs-derived peu-MIR2916 can downregulate TLR4 gene expression. **A** miRNA delivery detection: RT-PCR was used to detect the absorption of miRNA in normal and ALF mice after oral administration of *Artemisiae Argyi* juice (100 μl, 207 mg) and AELNVs (100 mg/kg). **B** miRNAs in the peripheral blood of mice with AELNVs administrated i.g. compared to models (n = 3 per group). **C** miRNAs in the liver of mice with AELNVs administrated i.g. compared to models (n = 3 per group). **D** Dual-luciferase reporter gene assays were performed to confirm the interaction between peu-MIR2916 and TLR4. Luciferase reporter gene plasmids containing a wild-type (TLR4-WT) or a mutated (TLR4-MU) 3′-UTR sequence were constructed and were co-transfected with miR2916 mimics or the corresponding control (miRNA-NC) into 293T cells. and Renilla and firefly fluorescence levels of cell lysis were detected. **E** The effect of miR2916 mimics on the mRNA levels of TLR4 was detected by RT-qPCR. HepG2 cells were pretreated with LPS (1 mg/ml) for 6 h. Then, AELNVs (5 μg/ml), miRNA-NC (50 nM), or miR2916 mimics (30 nM and 100 nM) were added and incubated for 12 h, then cells were collected for RT-qPCR assay (n = 3 per group). **F** Western blot analysis and quantitative results **G** of TLR4, NLRP3, NF-κB P65 and Nrf2 in HepG2 protein expression (n = 3 per group). Data represent means ± SEM. Statistical analysis was performed by one-way ANOVA. *p < 0.05, **p < 0.01, ***p < 0.001, ****p < 0.0001. ns, no significance

To verify the role of these miRNAs, we selected peu-MIR2916-like miRNAs: peu-MIR2916-p3_1ss12TC2 and peu-MIR2916-p5_1ss13TG, which represent two different binding modes with TLR4-3'UTR sequences, and named them miR2916-12 and miR2916-13, respectively. First, it is necessary to verify whether these miRNAs have been successfully delivered to the target cells, we detected the absorption of these two miRNAs in normal and ALF mice after oral administration of *Artemisiae Argyi* juice and AELNVs using RT-qPCR (Fig. 8A). The results showed that both were detected in the peripheral blood and liver of mice, demonstrating their effective delivery and accumulation (Fig. 8B, C). Compared with juice, mice orally administered with AELNVs showed better efficiency in miRNA delivery. In addition, it was found that there was a significant increase in both of them in the liver of ALF mice treated with AELNVs. Indicating that miRNAs derived from AELNVs are more likely to accumulate in large quantities in damaged liver.

To validate the direct targeting of TLR4 by AELNVs-derived miR-2916 (peu-MIR2916-p3_1ss12TC), a dual-luciferase reporter assay was performed. Co-transfection of 293 T cells with a plasmid containing the wild-type TLR4 3'UTR and peu-MIR2916 mimics resulted in a significant decrease in luciferase activity, an effect that was abolished when the binding site was mutated (Fig. 8D). Furthermore, transfection of peu-MIR2916 mimics into LPS-stimulated Huh7 cells led to a dose-dependent reduction in TLR4 mRNA levels (Fig. 8E). Western blot analysis demonstrated that peu-MIR2916 mimics suppressed the activation of the NF-κB pathway and NLRP3 inflammasome, while promoting nuclear translocation of Nrf2 (Fig. 8F, G). In line with these findings, both mRNA (Figure S3) and protein (Fig. 4B) levels of TLR4 were correspondingly decreased in the livers of ALF mice following AELNVs intervention. Collectively, these results confirm that AELNVs deliver peu-MIR2916 to target TLR4, thereby modulating the TLR4/NLRP3/Nrf2 signaling axis to activate autophagy and mitigate oxidative stress and inflammation.

## Discussion

Our previous research demonstrated that *Artemisiae Argyi* exosome-like nanovesicles effectively alleviated dextran sulfate sodium (DSS)-induced ulcerative colitis in animal models, restored the integrity of the intestinal barrier, and reversed the imbalance in gut microbiota composition [25]. Given the close interaction between the gut and liver through the "gut-liver axis," and considering that gut microbiota dysbiosis and barrier damage can lead to the translocation of endotoxins (such as lipopolysaccharide), thereby inducing intestinal endotoxemia and exacerbating liver injury, this study further investigated the role of AELNVs in acute liver failure. The experimental results demonstrated that AELNVs exhibited significant immediate protective effects against LPS/D-GalN-induced ALF in mice. Specifically, AELNVs inhibited inflammatory responses, alleviated oxidative stress, and reduced hepatocyte apoptosis, thereby effectively improving pathological symptoms and enhancing survival rates within a short period. It should be noted that although AELNVs have shown potential in modulating gut microbiota in models of intestinal inflammation, the potential impact on gut microbial composition was not the central focus of this study. Therefore, whether AELNVs exert their hepatoprotective effects by modulating the "gut-liver axis" remains to be systematically validated in subsequent experiments.

In the murine model of LPS/D-GalN-induced acute liver failure, the pathological process is fundamentally TLR4-dependent, highlighting its therapeutic relevance as a molecular target [24, 26]. Activation of TLR4 initiates a signaling cascade that engages the innate immune system through NF-κB transduction, subsequently provoking systemic inflammation via elevated pro-inflammatory cytokine production [27, 28]. At the hepatic level, this cascade stimulates macrophage recruitment and activation, accompanied by excessive generation of inflammatory mediators and reactive oxygen species, which collectively aggravate hepatocellular injury. These events progress to large-scale apoptosis and necrosis of primed hepatocytes, initiating a severe inflammatory outcome [1, 29]. Our investigation newly reveals that AELNVs administration substantially diminishes circulating concentrations of the pro-inflammatory factors TNF-α, IL-1β, IL-6, and IFN-γ in ALF mice, while concurrently inhibiting hepatocyte apoptosis, leading to overall hepatic improvement. This supports the conclusion that AELNVs can effectively moderate inflammatory mediator release and ameliorate the hepatic inflammatory milieu. Previous reports establish that modulation of the NLRP3 pathway in macrophages alleviates liver injury, including in ALI and ALF models [30, 31]. Transcriptional upregulation of NLRP3 is also known to follow TLR4 stimulation in hepatocytes and Kupffer cells [24]. Assembly of the NLRP3 inflammasome promotes Caspase-1 activation, resulting in massive production of pro-inflammatory cytokines [30]. Our data demonstrate that AELNVs significantly attenuated the activation of TLR4 and NLRP3, as well as the maturation of Caspase-1, compared to LPS/GalN-challenged animals. Furthermore, AELNVs elevated the expression of the autophagy-related proteins LC3-II and Beclin-1, while reducing p62 accumulation in mouse hepatocytes. These collective findings suggest that the hepatoprotective effect of AELNVs against LPS/GalN-induced injury operates through restraint of inflammatory signaling and concurrent restoration of autophagic activity.

Building on the aforementioned observations regarding core inflammatory pathways, we further investigated the immunomodulatory capacity of AELNVs in reprogramming the hepatic immune landscape. Our data revealed that AELNVs treatment notably elevated the population of restorative CD206⁺ M2-type macrophages while simultaneously reducing the prevalence of pro-inflammatory CD86⁺ M1-type macrophages in ALF mice. This phenotypic shift appears mechanistically linked to the previously documented induction of autophagy by AELNVs, as Kupffer cell-induced autophagy has been established as a pivotal driver promoting M2 polarization [32, 33]. Further characterization of macrophage surface markers confirmed substantial alterations in population composition. This rebalancing of immune homeostasis can be partially explained by the ability of AELNVs to curtail cellular recruitment: through downregulation of the monocyte chemoattractant CCL7, AELNVs limited the infiltration of monocyte-derived MoMFs from circulation into hepatic tissue [34]. By analyzing the chemokines in mouse liver tissue, we found that AELNVs also inhibited signal transduction on the CCL5-CCR5 and CXCL9/10/11-CXCR3 axes. These chemokines can regulate the differentiation and migration of different T cell subsets [35].

In the liver, cytochrome P450 (CYP) is the main endoplasmic reticulum membrane-bound hemoglobin. It maintains the balance level of bilirubin, controls endoplasmic reticulum stress and oxidative stress, which is crucial for ensuring cell survival [36, 37]. Many previous studies have shown that mouse liver CYP2A5 and its human homolog CYP2A6 catalyze the metabolism of many drugs and toxins, and Cyp2A5 directly regulates heme and its metabolite bilirubin (BR) [18, 38, 39]. Although heme is crucial for cellular metabolism involving oxygen transfer, it can produce reactive oxygen species and exhibit cytotoxicity at high concentrations, leading to apoptosis of primary liver cells. Therefore, it is necessary to strictly regulate heme and BR to prevent heme/BR related cytotoxicity and protect cells from the invasion of oxygen free radicals, Cyp2A5 can oxidize BR to the less toxic intermediate biliverdin (BV) in the liver [38, 40]. The expression of CYP2A5 is strictly controlled by Nrf2 in the liver, and Nrf2 mediated Cyp2a5 induction protects mouse liver cells from reducing endoplasmic reticulum stress. Therefore, upregulation of Cyp2a5 is a useful indicator of liver activation in the Nrf2 pathway. When oxidative stress occurs, Nrf2 is released from the Keap1 binding inhibition complex in the cytoplasm and transferred to the nucleus, activating the expression of antioxidant genes [40, 41]. TLR signaling regulates the antioxidant Nrf2 signaling pathway. Our study found that AELNVs can interfere with TLR4/NF-κB signaling, downregulate Keap1, and regulate Nrf2 to promote CYP2A5 transcription.

Interestingly, while the function of most other CYP enzymes is impaired, CYP2A5 is induced under pathological conditions associated with liver injury and remains unchanged during the chronic phase of mouse schistosomiasis [42], but is upregulated in malari, viral hepatitis, fastin, bacterial hepatitis, and liver cancer [43]. Our study shows that CYP2A5 is significantly downregulated in LPS/D-GalN-induced ALF, suggesting a distinct gene regulatory pattern under acute inflammatory conditions. CYP2E1 is widely recognized as a key contributor to ethanol-induced oxidative stress, ethanol-induced liver injury, and alcoholic fatty liver disease [44]. Ethanol-induced CYP2E1 co-localizes with CYP2A5 and is upregulated prior to CYP2A5, with both enzymes being regulated by Nrf2 [44, 45]. In this study, we found that CYP2E1 expression was unaffected in LPS/D-GalN-induced ALF mice, and AELNVs did not promote its expression (Fig. 5E), indicating that Nrf2 exerts differential regulatory effects on various CYP enzymes depending on the pathological context. The precise mechanism by which Nrf2 regulates Cyp2A5 gene expression is unclear. Knockout mice of related genes will be helpful for further research.

PELNVs has multiple advantages, including cycle stability, high biocompatibility, biodegradability, low immunogenicity, scalability, and the ability to enrich bioactive components (such as lipids, proteins, and miRNA), making it a very promising drug delivery platform. MiRNAs derived from PELNVs have recently garnered significant research interest as innovative bioactive constituents capable of mediating cross-kingdom RNA interference in eukaryotic organisms. Dietary and medicinal PELNVs-derived miRNAs can be internalized by intestinal epithelial cells via the S1DT1 transporter, subsequently reaching systemic circulation to modulate endogenous gene expression in mammalian systems [46]. Functionally, PELNVs-derived miRNAs demonstrate considerable therapeutic promise: in a representative study, miR-396e encapsulated in garlic-derived extracellular vesicles was shown to ameliorate non-alcoholic fatty liver disease by targeting PFKFB3 and attenuating macrophage-driven inflammation [47]. Similarly, extracellular nanoparticles from Cc-ELNs delivered miRNA-5106 to restore zinc homeostasis and suppress neutrophil extracellular trap formation, thereby alleviating colitis [48]. A key feature underpinning these effects is the structural resilience of PELNVs-derived miRNAs, attributed to 3'-terminal 2-O-methylation modifications that confer remarkable resistance to oxidative stress, enzymatic degradation, and harsh gastrointestinal conditions. This biochemical robustness highlights the translational potential of PELNVs-derived miRNAs as both therapeutic agents and nutraceutical supplements for managing human pathologies [49]. Our data indicate that miRNAs in AELNVs exert hepatoprotective effects by inhibiting the gene expression of TLR4 in ALF mouse liver tissue. Interestingly, AELNV contains multiple miRNAs with peu-MIR2916-like sequences that inhibit TLR4 gene expression, strongly bind to the 3'UTR sequence of TLR4, and exhibit two different base pairing structures. The RT qPCR results demonstrated the effective delivery and accumulation of miRNAs in AELNVs in damaged liver. Compared with juice, mice orally administered with AELNVs showed better efficiency in miRNA delivery. These results further demonstrate that MIR2916 is a key component of AELNVs in treating ALF.

## Conclusion

This study identifies and characterizes extracellular vesicle-like nanoparticles derived from *Artemisiae Argyi* (AELNVs) as a promising therapeutic candidate against acute liver failure. We demonstrate that orally administered AELNVs enhance survival in LPS/D-GalN-induced ALF mice by attenuating hepatic injury through multiple mechanisms: curtailing the release of inflammatory mediators, inhibiting apoptotic signaling, and inducing autophagy. Mechanistically, the hepatoprotective effect is largely attributed to MIR2916 contained within AELNVs, which coordinately modulates the TLR4/NLRP3/Nrf2 signaling axis. These findings position AELNVs as a potential treatment option for clinical liver disease management.

## Supplementary Information

Supplementary material 1.

## Data Availability

The data sets, analytical procedures and experimental details used in the current study are available from the corresponding author upon reasonable request.

## Abbreviations

AELNVs: Exosome-like nanovesicles derived from Artemisia argyi
ALF: Acute liver failure
DAMPs: Damage-associated molecular patterns
PELNVs: Plant-derived extracellular vesicle-like nanovesicles
EPR: Enhanced permeability and retention
Nrf2: Nuclear factor erythroid 2-related factor 2
RPS: Resistive pulse sensing
DLS: Dynamic light scattering
DAB: Diaminobenzidine
ALT: Alanine aminotransferase
AST: Aspartate aminotransferase
LDH: Lactate dehydrogenase
GSH: Glutathione
GPx: Glutathione peroxidase
MPO: Myeloperoxidase
MDA: Malondialdehyde
TFF: Tangential Flow Filtration
TEM: Transmission electron microscopy
PCA: Principal Component Analysis
GO: Gene Ontology analysis
KEGG: Kyoto Encyclopedia of Genes and Genomes
ROS: Reactive oxygen species
CYP2A5: Cytochrome P450 2A5
HO-1: Heme oxygenase-1
CCR5: C-chemokine receptor 5
MoMFs: Monocyte-derived macrophages
KCs: Kupffer cells
BR: Bilirubin
BV: Biliverdin
