# Supplementary material for: Artemisiae Argyi exosome-like nanovesicles alleviate ALF by reducing oxidative stress and inhibiting inflammation through the TLR4/NLRP3/Nrf2 axis
Source: Chin Med. 2026 Feb 16;21:69. doi: 10.1186/s13020-026-01345-9 (PMC12911172; doi:10.1186/s13020-026-01345-9)
Supplement: Supplementary file 1 — Supplementary material 1. [file 13020_2026_1345_MOESM1_ESM.docx]

# Supplementary Materials

***Artemisiae Argyi* exosome-like nanovesicles alleviate ALF by reducing oxidative stress and inhibiting inflammation through the TLR4/NLRP3/Nrf2 axis**

Wenjie Zheng^1,2,3,#^, Yue Su^1,2,3,#^, Yan Tang^1,2,3^, Kexin Yu^2,3^, Runlin Lin^2,3^, Yiyi Shan^2,3^, Yuanyuan Wang^2,3^, Louqin Fu^2,3^, Jingjing Li^2,3^*


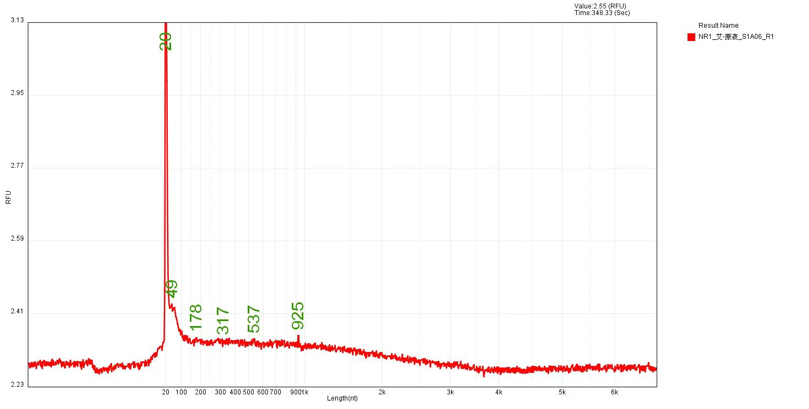
**Figure S1**：RNA composition in AELNVs was analyzed using the BIO Fragment Analyzer.


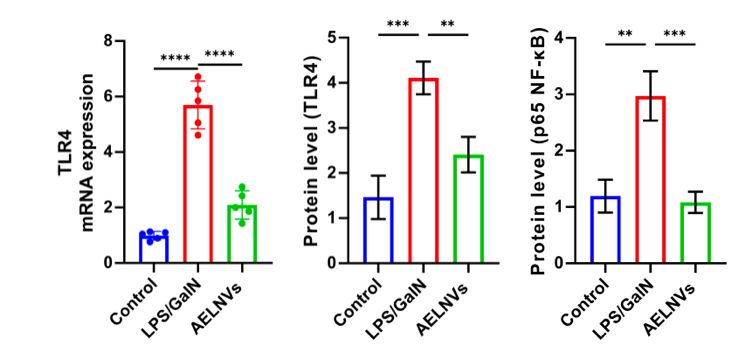
**Figure S2**：*In vivo* whole-body imaging to detect the distribution of AELNVs in mice at 0, 1, 2, 4, and 6 hours after oral administration.


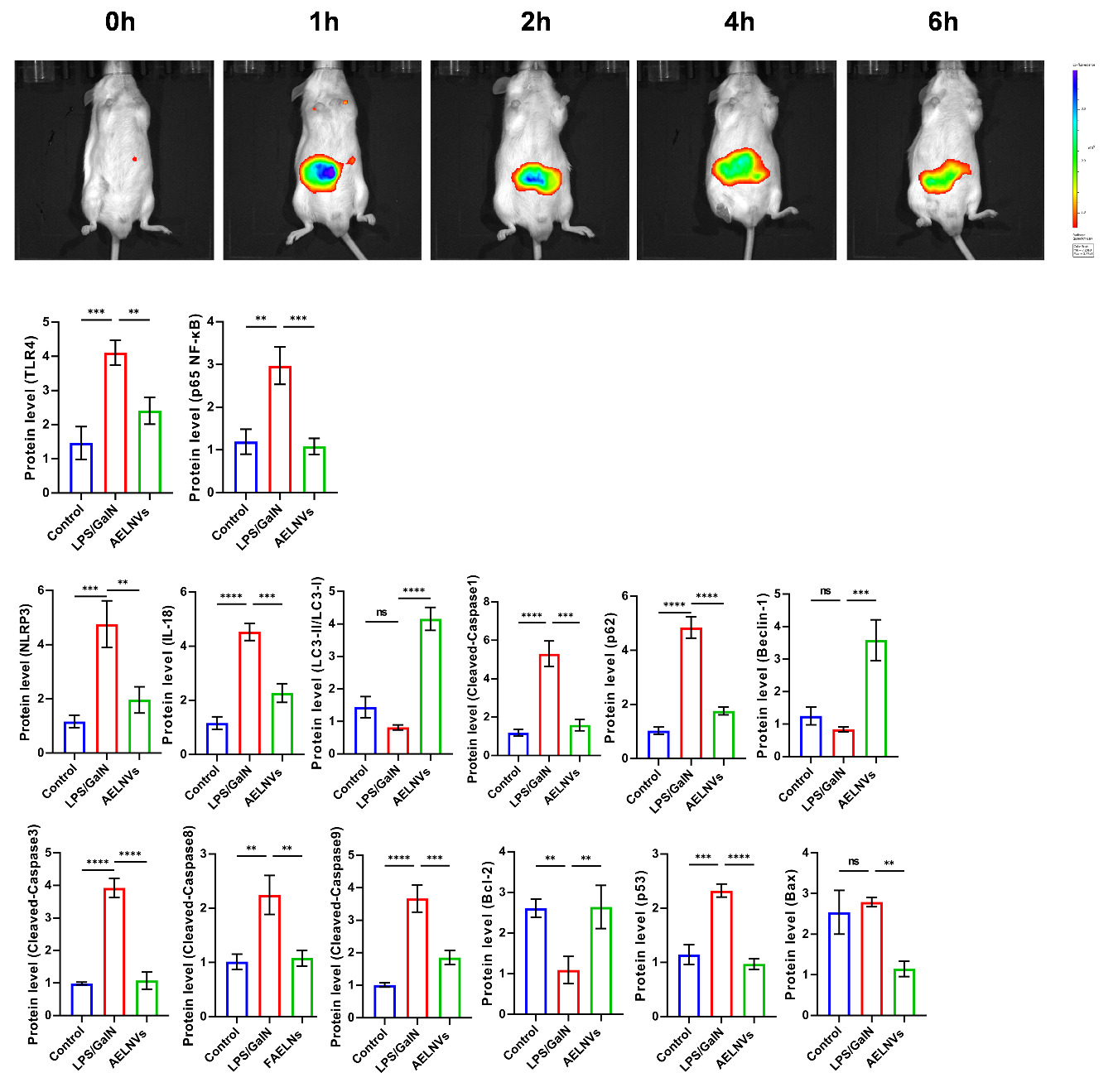
**Figure S3**：The mRNA expression of TLR4 in the liver of mice and quantitative Western blot analysis was performed using ImageJ software to determine the band intensities of TLR4 and NF-κB p65 protein expression TLR4. Data represent means ± SEM. Statistical analysis was performed by one-way ANOVA. *p < 0.05, **p < 0.01, ***p < 0.001, ****p < 0.0001.


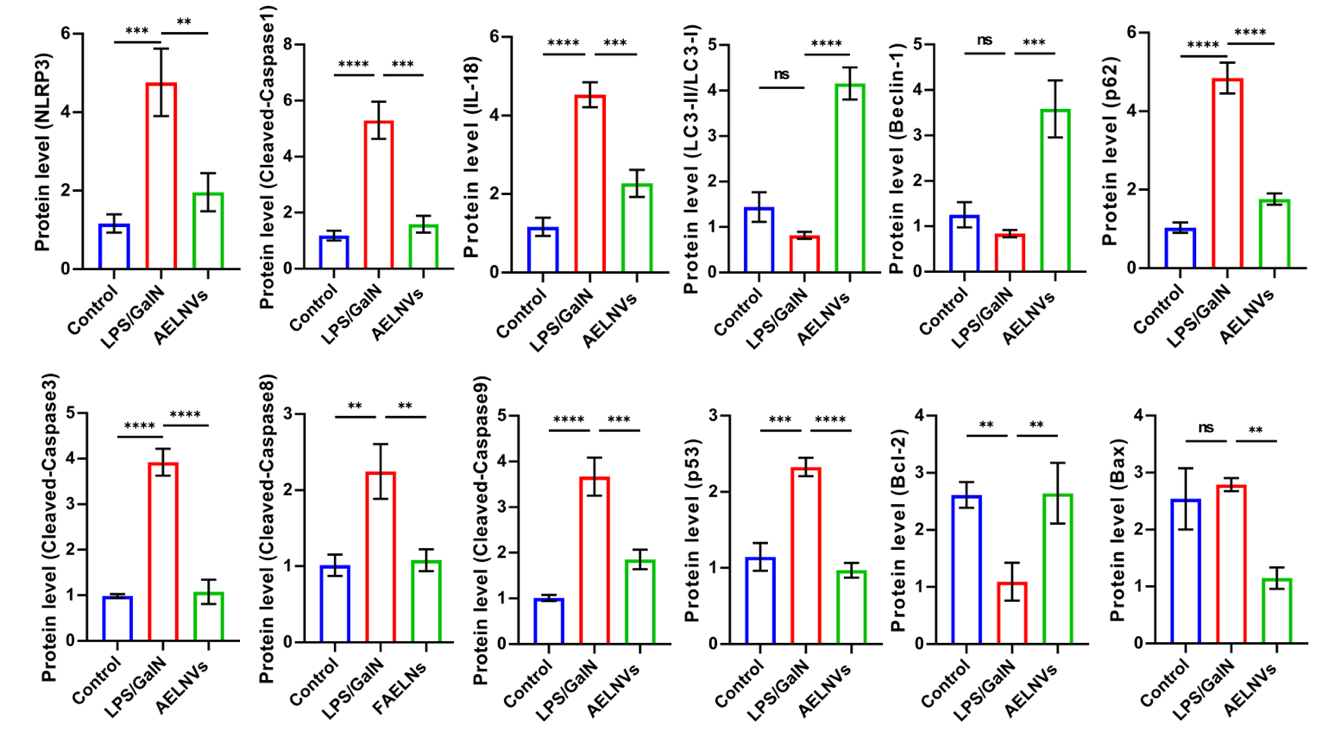
**Figure S4**：Quantitative Western blot analysis was performed using ImageJ software to determine the band intensities of NLRP3, Caspase1, IL18, LC3-I, LC3-II, Beclin-1and p62 protein expression. Data represent means ± SEM. Statistical analysis was performed by one-way ANOVA. *p < 0.05, **p < 0.01, ***p < 0.001, ****p < 0.0001.


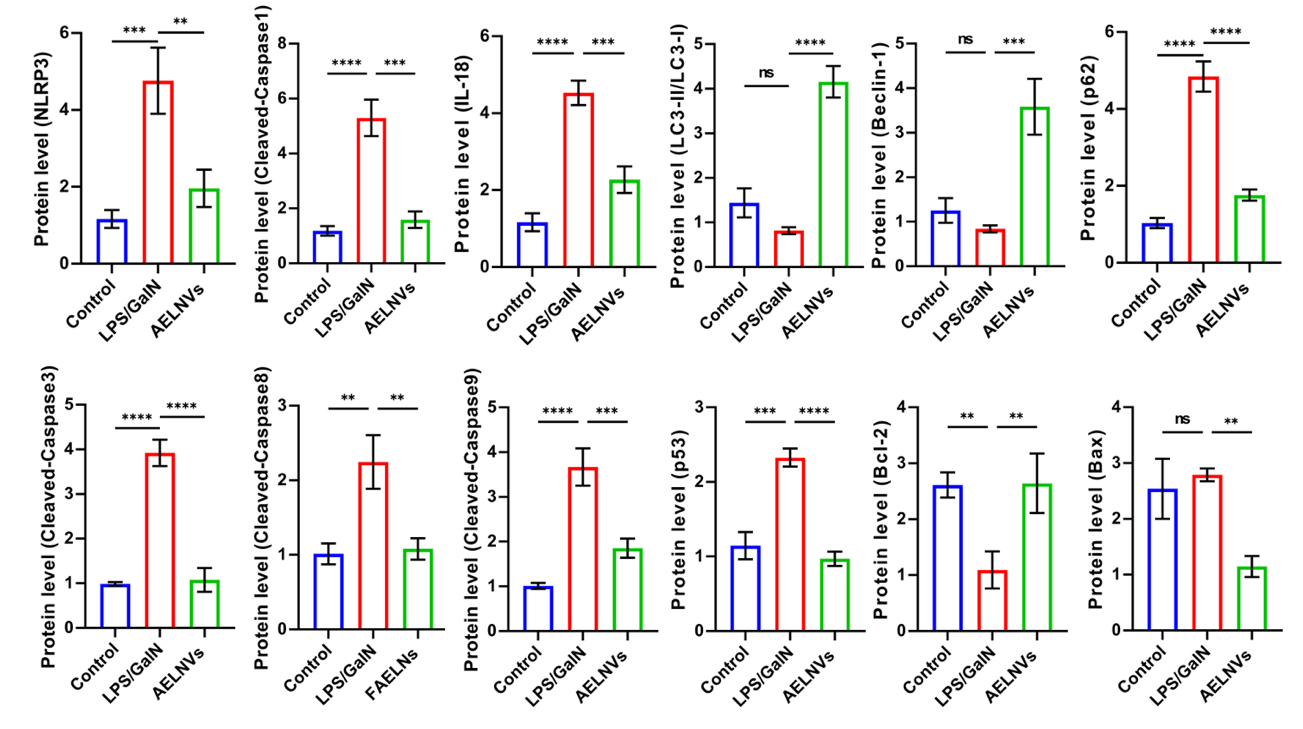
**Figure S5**：Quantitative Western blot analysis was performed using ImageJ software to determine the band intensities of Caspase3, Caspase8, Caspase9, p53, Bcl2 and Bax protein expression. Data represent means ± SEM. Statistical analysis was performed by one-way ANOVA. *p < 0.05, **p < 0.01, ***p < 0.001, ****p < 0.0001.

**
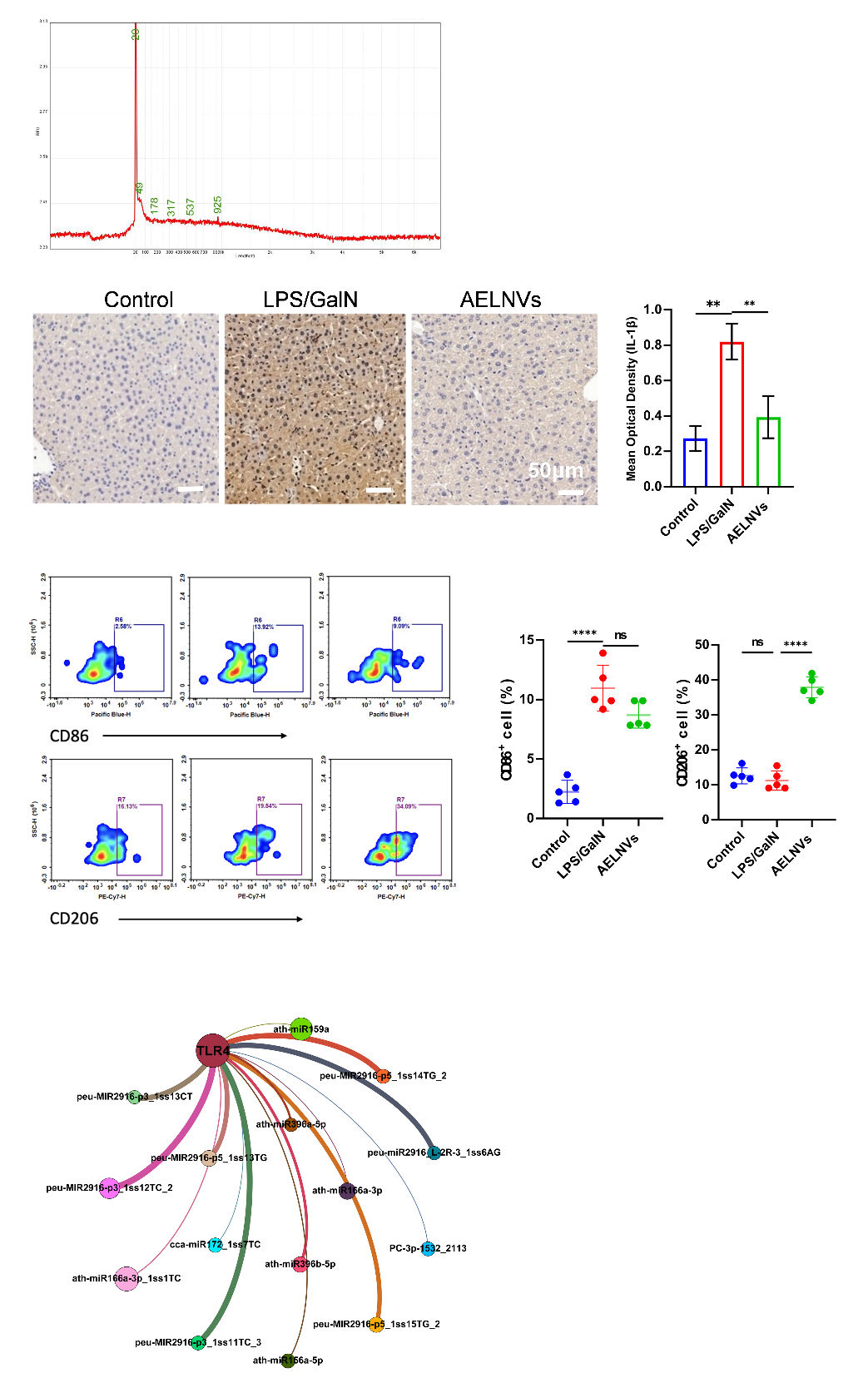
Figure S6**：Immunohistochemical analysis of IL-1β in the liver (n = 3 per group). Scale bar: 50 μm.

IL-1β


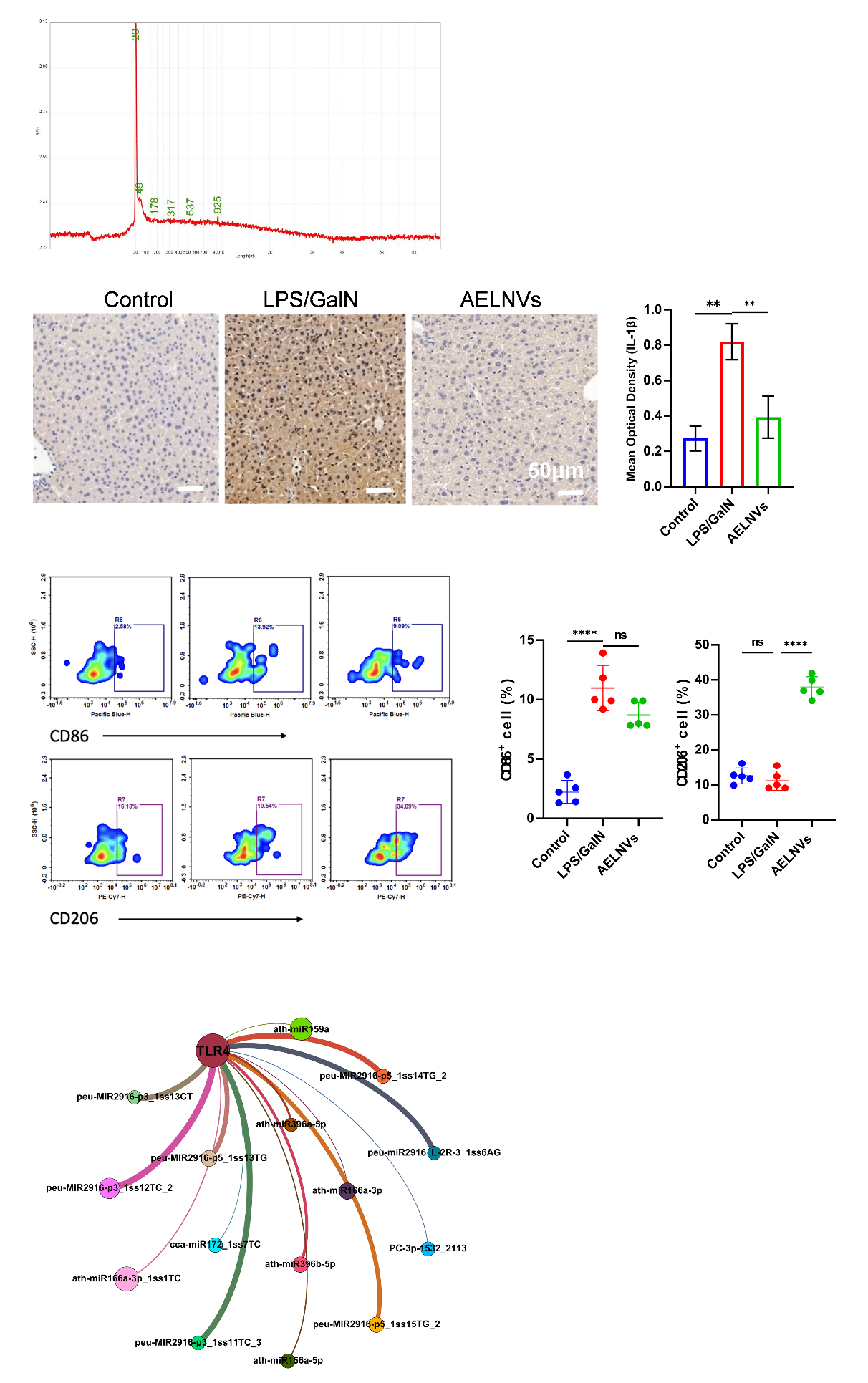
**Figure S7**：Content of M1/M2 macrophages in the liver analyzed by flow cytometry, CD86^+^resident M1 macrophages, CD206^+^ resident M2 macrophages (n = 5 per group).


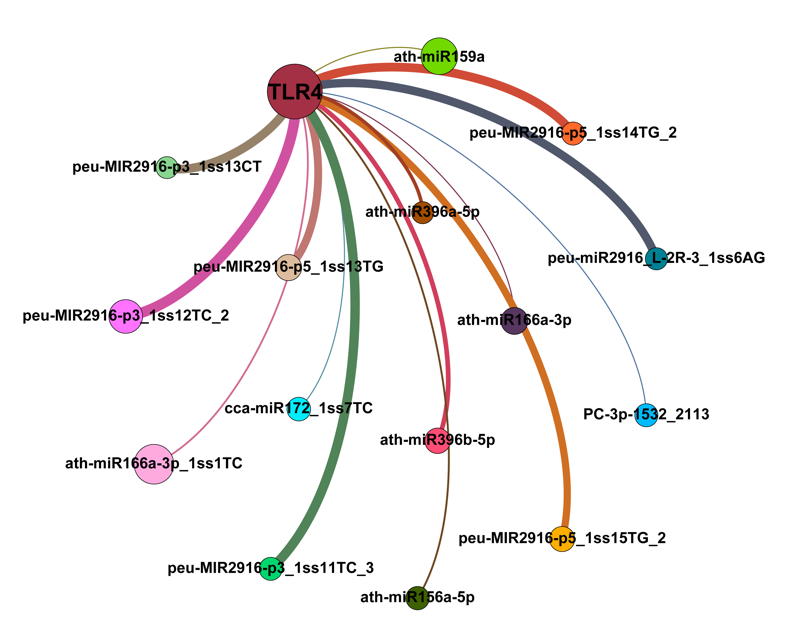
**Figure S8**：Gene regulatory network diagram indicates that the TLR4 gene is significantly correlated with peu MIR2916-like sequences in the top 15% of miRNAs expressed in AELNVs, the thickness of the lines represents the degree of correlation.

**TABLE 1.**Sequence of primers used for RT-qPCR assay.

| gene | Forward Sequence | Reverse Sequence |
| --- | --- | --- |
| CCL5(mouse) | GCTGCTTTGCCTACCTCTCC | TCGAGTGACAAACACGACTGC |
| CCR5(mouse) | TGGGGTGGAGGAGCAGGGAG | TAGGCCACAGCATCGGCCCT |
| CCL7(mouse) | TCTACGCAGTGCTTCTTTGCC | AAGGGGGATCTTCAGCTTTAGTA |
| CXCR3(mouse) | AGCCATGTACCTTGAGGTTAG | GGTTCTGTCAAAGTTCAGGC |
| CXCL9(mouse) | GCAACAAAACTGAAATCATTGCT | ATTCAGGGTGCTTGTTGGTA |
| CXCL10(mouse) | TGTTGAGATCATTGCCACG | GAAGACCAAGGGCAATTAGG |
| CXCL11(mouse) | CTCTGTGCAAACCTAACCCC | CCTCTTTTGGTCAGGAATACCAC |
| Cyp2a5(mouse) | GGACAAAGAGTTCCTGTCACTGCTTC | GTGTTCCACTTTCTTGGTTATGAAGTCC |
| Keap1 (mouse) | CCTGCACAACTGTATCTATGCT | CTGTATCTGGGTCGTAACACTCC |
| TLR4 (mouse) | ACTTTATCCAACCAGGTGCAT | GCTAATCGGAATTCTTCAATGGTC |
| HO-1(mouse) | CACTCTGGAGATGACACCTGAG | GTGTTCCTCTGTCAGCATCACC |
| GPX4(mouse) | CCTCTGCTGCAAGAGCCTCCC | CTTATCCAGGCAGACCATGTGC |
| GAPDH(mouse) | ACAGTCCATGCCATCACTGCC | GCCTGCTTCACCACCTTCTTG |
| peu-MIR2916-p3_1ss12TC2 | CCGACCAGGGATCGGC | ACTGCAGGGTCCGAGGT |
| peu-MIR2916-p5_1ss13TG | GCGGCCGAAGACGATCA | ACTGCAGGGTCCGAGGT |

| Antibody | Brand | Cat No. | Species | Dilution ratio | Incubation conditions |
| --- | --- | --- | --- | --- | --- |
| TLR4 | Proteintech | 19811-1-AP | Human, Mouse, Rat and More | 1:1000 | 4°C, overnight |
| NF-κB p65 | Cell Signaling | 8242S | Human, Mouse, Rat and More | 1:1000 | 4°C, overnight |
| NLRP3 | Cell Signaling | 15101S | Human, Mouse | 1:1000 | 4°C, overnight |
| Caspase1 | Cell Signaling | 83383T | Human, Mouse, Rat | 1:1000 | 4°C, overnight |
| IL18 | Proteintech | 33710-1-AP | Mouse | 1:5000 | 4°C, overnight |
| LC3 | Cell Signaling | 2775S | Human, Mouse, Rat | 1:1000 | 4°C, overnight |
| Beclin-1 | Cell Signaling | 3495S | Human, Mouse, Rat and Monkey | 1:1000 | 4°C, overnight |
| p62 | Proteintech | 84826-1-RR | Human, Mouse, Rat | 1:5000 | 4°C, overnight |
| Caspase3 | Cell Signaling | 9662S | Human, Mouse, Rat and More | 1:1000 | 4°C, overnight |
| Caspase8 | Cell Signaling | 4790S | Human, Mouse, Rat | 1:1000 | 4°C, overnight |
| Caspase9 | Cell Signaling | 9508S | Human, Mouse, Rat and More | 1:1000 | 4°C, overnight |
| p53 | Cell Signaling | 2524S | Human, Mouse, Rat and More | 1:1000 | 4°C, overnight |
| Bcl2 | Cell Signaling | 3498S | Human, Mouse | 1:1000 | 4°C, overnight |
| Bax | Cell Signaling | 2772S | Human, Mouse, Rat and Monkey | 1:1000 | 4°C, overnight |
| Cyp2A5 | Aladdin | Ab098678 | Human, Mouse | 1:2000 | 4°C, overnight |
| Cyp2E1 | Invitrogen | PA5-79132 | Human, Mouse, Rat | 1:1000 | 4°C, overnight |
| Nrf2 | Proteintech | 66504-1-Ig | Human, Mouse, Rat and More | 1:5000 | 4°C, overnight |
| β-tublin | Cell Signaling | 2128S | Human, Mouse, Rat and More | 1:1000 | 4°C, overnight |

**TABLE 2.**Information for all antibodies used in the Western blot experiments
